# Bispecific NK‐Cell Engager Targeting TP53 Mutation Through Integration of a TCR‐mimic Antibody

**DOI:** 10.1002/mco2.71058

**Published:** 2026-10-15

**Authors:** Hanfei Wu, Zhe Zhao, Li Zhang, Xuan Xiong, Mengdie Yang, Weiping Ge, Danyue Zhi, Xiaobin Cao, Chuanqian Yi, Yong Qiao, Junjun Cao, Yan Mei, Shiping Jiao, Quan Liu, Hao Zhou, Zhongjuan Xu, Guangli Suo

**Affiliations:** ^1^ School of Nano‐Tech and Nano‐Bionics University of Science and Technology of China Hefei Anhui China; ^2^ CAS Key Laboratory of Nano‐Bio Interface Suzhou Institute of Nano‐Tech and Nano‐Bionics Chinese Academy of Sciences Jiangsu China; ^3^ Department of Pharmaceutical Engineering School of Engineering China Pharmaceutical University Nanjing Jiangsu China; ^4^ Department of Vaccine Clinical Evaluation Jiangsu Provincial Center for Disease Prevention and Control Nanjing China; ^5^ Nanjing LivingChip Biotechnology Co., Ltd Nanjing China; ^6^ Clinical Research Ward The Sixth People's Hospital Shanghai Jiao Tong University Shanghai China; ^7^ TCRX Therapeutics (KeShiHua) Co. Ltd Nanjing China; ^8^ Department of Otolaryngology Eye, Ear, Nose and Throat Hospital Fudan University Shanghai China; ^9^ Skull Base Adenoid Cystic Carcinoma Center Eye, Ear, Nose and Throat Hospital Fudan University Shanghai China; ^10^ Department of General Surgery The First Affiliated Hospital of Soochow University Suzhou China

**Keywords:** neoantigen, NK cell engager, NK‐cell‐mediated immunotherapy, single‐chain diabody, TCR‐mimic antibody, TP53 mutation

## Abstract

Natural killer (NK) cells are valuable for cancer immunotherapy because they kill tumor cells without prior sensitization or human leukocyte antigen (HLA) restriction. However, their efficacy is often limited by immune‐evasion, especially major histocompatibility complex I (MHC‐I)‐mediated NK cell inhibition. NK cell engagers (NKCEs) can redirect NK cells to tumor for more effective immunotherapy, but most current NKCEs target tumor‐associated antigens (TAAs), risking on‐target, off‐tumor toxicity in clinical applications. Neoantigens, as tumor‐specific antigens (TSAs) arising from somatic mutations and presented by MHC‐I on tumor cell surfaces, represent ideal tumor‐specific target. Here, we engineered NKCEs built on neoantigen‐targeting TCR‐mimic antibodies (TCRm‐Abs) to enhance tumor‐specific NK cell‐mediated cytotoxicity. Using a high‐throughput hydrogel‐based microwell single‐cell chip (HMSC‐chip) coupled with T2 cell‐based de‐selection, we identified C33‐Ab, a functional TCRm‐Ab specific for p53^R175H^/HLA‐A*02:01, from ∼500,000 hybridoma cells within 8 weeks. Based on this TCRm‐Ab, we constructed a bispecific NKCE (C33×CD16), a single‐chain diabody (scDb) linking C33‐Ab to an anti‐CD16 scFv. C33×CD16 specifically bridges NK cells with p53^R175H^/HLA‐A*02:01‐positive cancer cells. It potently enhanced NK cell‐mediated cytotoxicity in vitro and antitumor efficacy in vivo upon adoptive NK‐cell transfer. This TCRm‐Ab‐based bispecific NKCE offers a promising strategy for tumor‐specific NK cell‐mediated immunotherapy by targeting intracellular oncogenic hotspot mutations.

## Graphical abstract

Most existing NK cell engagers (NKCEs) target tumor‐associated antigens (TAAs), risking on‐target, off‐tumor toxicity. We engineered a novel NKCE termed C33×CD16, a single‐chain diabody (scDb) constructed by fusing TCR‐mimic antibody C33‐Ab (targeting p53^R175H^/HLA‐A*02:01) to the scFv of 3G8 (anti‐CD16 antibody) (a). C33×CD16 specifically bridges NK cells with cancer cells expressing p53^R175H^/HLA‐A*02:01 (b), offering a promising strategy for tumor‐specific NK cell‐mediated immunotherapy by targeting intracellular oncogenic hotspot mutations.

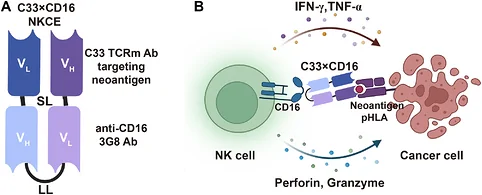

## 1 Introduction

Natural killer (NK) cells are cytotoxic lymphocytes of the innate immune system that can kill cancerous cells without prior sensitization or human leukocyte antigen (HLA) restriction, positioning NK cell‐based cancer therapy as a crucial innovation in immunotherapy [1, 2]. However, immune‐evasion mechanisms often impair NK cell antitumor activity, posing a significant challenge to the development of effective NK cell‐based therapies [3]. To overcome this limitation, NK cell engagers (NKCEs), a class of multi‐specific immunotherapeutic molecules designed to redirect NK cells toward tumor cells, have recently been developed as promising tools for precision cancer immunotherapy [4, 5, 6].

NKCEs offer advantages over bispecific T‐cell engagers (BiTEs) in immunotherapy, including a reduced risk of severe cytokine release syndrome (CRS) [7, 8, 9, 10], efficacy against HLA‐loss tumors [11, 12], and compatibility with allogeneic NK cell‐based therapies [13, 14]. However, tumor‐associated antigens (TAAs) expression in normal tissues may cause on‐target, off‐tumor toxicity. Moreover, although downregulation of class I HLA (also known as MHC‐I) in tumors facilitates T cell evasion, many tumors retain elevated MHC‐I expression to engage NK cell inhibitory receptors such as KIRs and NKG2A, directly inhibiting NK cell activity [15, 16, 17, 18, 19].

To address these limitations, we propose that NKCEs can be developed using T cell receptor‐mimic antibodies (TCRm‐Abs) targeting MHC‐I‐presented neoantigens to enhance tumor specific NK cell‐mediated cytotoxicity. Neoantigens, a type of tumor‐specific antigen (TSA), are abnormal peptides derived from somatic mutations in tumor cells [20, 21]. Neoantigens are presented on the cancer cell surface by HLA as peptide‐HLA (pHLA) complexes [22, 23], which can trigger immune responses. Therefore, neoantigens represent ideal targets for precision immunotherapy due to their tumor specificity and their ability to convert intracellular mutations into extracellularly accessible targets. Although neoantigen‐targeting TCRm‐Abs have recently been employed in the development of BiTEs to improve cancer‐specific immunotherapy [24, 25, 26], their application in NKCE design remains unexplored.

*TP53* encodes the intracellular tumor suppressor protein p53 and is frequently mutated in the majority of human tumors [27, 28, 29, 30, 31, 32]. The p53^R175H^ mutation, characterized by an arginine‐to‐histidine substitution at position 175 [33], produces the neoantigen HMTEVVRHC (mutant amino acid underlined), forming the p53^R175H^/HLA‐A*02:01 complex [24, 34, 35], thereby transforming intracellular mutations into extracellularly accessible targets. Due to its high prevalence across various cancers, TCRm‐Abs targeting p53^R175H^/HLA‐A*02:01 have been successfully discovered and used in BiTE design to specifically eliminate target cancer cells [24, 36, 37, 38, 39, 40].

Here, we report the development and therapeutic potential of a novel TCRm‐Ab‐based NKCE. We first established an efficient method for TCRm‐Ab discovery through high‐throughput screening of single hybridomas on hydrogel‐based microwell single‐cell chip (HMSC‐chip), followed by de‐selection based on T2 cell binding assay to exclude antibodies lacking neoantigen specificity. We initially isolated 308 single hybridoma cells that produced antigen pHLA‐specific antibodies by screening approximately half a million hybridomas arrayed on one HMSC‐chip. An optimal TCRm‐Ab (C33‐Ab), specifically recognizing p53^R175H^/HLA‐A*02:01, was finally successfully identified and engineered to a novel bispecific NKCE, C33×CD16, in the form of a single‐chain diabody (scDb). This scDb comprises one scFv derived from C33‐Ab and another scFv from the anti‐CD16 antibody 3G8 [41, 42], enabling dual binding to NK cells and cancer cells expressing p53^R175H^/HLA‐A*02:01, and amplifying NK cytotoxicity. Our results showed that C33×CD16 significantly enhances NK cell‐mediated tumor cytotoxicity in vitro and in vivo. This study presents a novel TCRm‐Ab‐based bispecific NKCE that improves NK cell activation targeting intracellular oncogenic hotspot mutations through neoantigens, thereby offering a promising strategy for tumor‐specific NK cell‐mediated immunotherapy.

## 2 Results

### 2.1 Discovery of Potential TCRm‐Abs Based on HMSC‐chip and T2 Cell Binding Assay

We discovered potential TCRm‐Abs through a high‐throughput method that combines single hybridoma screening using HMSC‐chip (Figure 1A) followed by de‐selection via T2 cell binding assay. Briefly, BALB/c mice were immunized with pHLA (p53^R175H^/HLA‐A*02:01) antigen to generate hybridomas. The resulting hybridomas were loaded onto the HMSC‐chips (LivingChip, China) to form a single cell array and cultured in medium supplemented with AF488‐p53^R175H^/HLA‐A*02:01 (green). Single hybridoma cells secreting antibodies targeting the p53^R175H^/HLA‐A*02:01 complex were identified on HMSC‐chip and precisely retrieved using an automated single‐cell selection system, CytoStar (LivingChip, China). The selected single hybridomas were then cultured in individual wells of a 96‐well plate for clonal growth. The secreted mAbs from each cell clone were collected for further selection of potential TCRm‐Ab by de‐selection with wild‐type (WT) p53 pHLA (p53^WT175^/HLA‐A*02:01) through T2 cell binding assay to eliminate the cross‐reactive clones.

**FIGURE 1:**
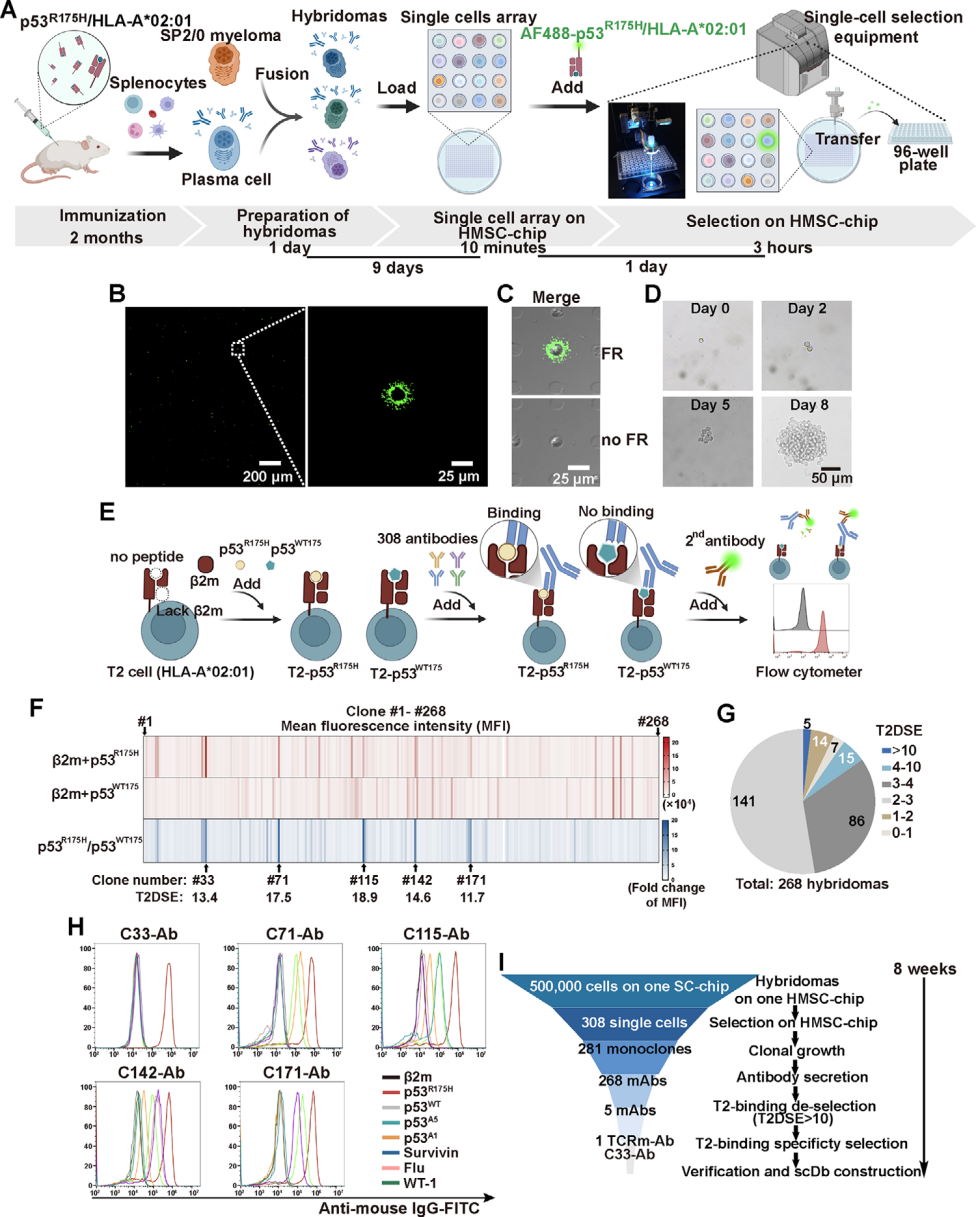
TCRm‐Ab screening using HMSC‐chip followed by T2 binding‐based de‐selection. (A) Schematic illustration of high‐throughput screening of pHLA (p53^R175H^/HLA‐A*02:01)‐targeting antibodies from single hybridoma cells using HMSC‐chip. Created in BioRender. Wu, H. (2026) https://BioRender.com/7scvnez. Representative HMSC‐chip fields where the FR indicates the pHLA‐targeting antibody secretion from a single hybridoma cell, displayed as IF images (B) and merged IF and bright‐field images (C). (D) Clone‐forming process of a single hybridoma cell retrieved from HMSC‐chip. (E) Schematic of de‐selection based on T2 binding assay using wild‐type control pHLA (p53^WT175^/HLA‐A*02:01). (F) Heatmap of T2 binding‐based de‐selection results. Red map: the MFI of β2m+p53^R175H^ or β2m+p53^WT175^ respectively; Blue map: the fold changes of the MFI of β2m+p53^R175H^ to β2m+p53^WT175^ (T2DSE). (G) Pie chart showing distribution of T2DSEs among mAbs from 268 hybridoma cells. (H) Cross‐reactivity of antigen peptides for five selected hybridoma clones (T2DSE > 10) through T2 binding assay. (I) Overview of TCRm‐Ab discovery combining HMSC‐chip and T2 binding‐based de‐selection.

The HMSC‐chip covers an area of 4 × 4 cm^2^ and contains approximately 547,600 microwells (18 µm diameter, 36 µm pitch and 20 µm depth). Each microwell is designed to accommodate only one hybridoma cell (Figure S1A,B). The hydrogel substrate of HMSC‐chip enables capture of secreted antibodies from single hybridomas and facilitates in situ antigen‐antibody reaction detectable by immunofluorescence (IF) assay. In this study, about 5 × 10^5^ hybridoma cells were loaded onto an HMSC‐chip to form single‐cell array within 10 min and cultured in medium simultaneously supplemented with AF488‐p53^R175H^/HLA‐A*02:01 (green). After 3 h of culture, fluorescent rings (FRs) surrounding some single hybridoma cells were observed on the HMSC‐chip, indicating local accumulation of anti‐p53^R175H^/HLA‐A*02:01 antibodies secreted by single live hybridoma cells (Figure 1B,C). We found that approximately 0.3% of hybridoma cells secreted antibodies that bind to AF488‐p53^R175H^/HLA‐A*02:01. We randomly selected 308 single hybridoma cells exhibiting green FRs from HMSC‐chip using CytoStar. Of these, 281 hybridoma cells formed clones after 8 days of culture, and 268 clones maintained proliferation and antibody secretion (Figure 1D). The supernatants of all these 268 hybridoma clones were performed de‐selection using T2 cells bearing either p53^R175H^/HLA‐A*02:01 (T2‐p53^R175H^) or control‐pHLA p53^WT175^/HLA‐A*02:01 (T2‐p53^WT175^) (Figure 1E). Potential TCRm‐Abs were selected based on T2 binding‐based de‐selection effectiveness (T2DSE), defined as the fold changes of the median fluorescence intensity (MFI) of T2 with defined surface targets (p53^R175H^/HLA‐A*02:01 to p53^WT175^/HLA‐A*02:01). T2DSE values were calculated and visualized in a heatmap (Figure 1F). Here, T2DSE functions as a benchmark for the preliminary identification of potential TCRm‐Abs. According to the result of a recent study, regarding anti‐p53^R175H^/HLA TCRm‐Ab generated using phage display method, phage clones with a T2DSE of p53^R175H^ to p53^WT175^ more than 4 were regarded as potential TCRm‐Abs [24]. In this study, we used a more stringent threshold of T2DSE ≥10 for potential TCRm‐Ab selection. Only 5 out of 268 clones (about 1.9%) met this criterion (Figure 1G), indicating the rarity of TCRm‐Ab‐secreting hybridomas within the antibody repertoire. These hybridoma clones (C33, C71, C115, C142, C171) were expanded, sequenced, and their supernatants were pooled for antibody purification via protein G‐agarose affinity chromatography.

### 2.2 Cross‐reactivity Test for TCRm‐Abs Against p53^R175H^/HLA‐A*02:01

To further evaluate the specificity of the five potential TCRm‐Abs we initially selected out based on HMSC‐chip and de‐selection by T2 cells assay, we conducted cross‐binding analysis using T2 cells pulsed with HLA‐A*02:01‐binding peptides to stabilize peptide/HLA‐A*02:01 (pHLA) complexes on the cell surface. In this analysis, three p53^R175H^ analog peptides (p53^WT175^, p53^A1^, p53^A5^) and three unrelated peptides (WT‐1, survivin, and Flu) were tested (Table S1 for peptide sequences). The p53^R175H^ analogs were designed with amino acid substitutions to minimize nonspecific binding (p53^A1^: the first histidine was substituted with alanine; p53^A5^: the valine at fifth loci was substituted with alanine), while the unrelated peptides served as controls to evaluate potential cross‐reactivity. Among these five tested mAbs, C33 mAb (C33‐Ab) displayed strong specificity to target T2‐ p53^R175H^ (Figure 1H, Figure S2A), with an 83.3% binding rate and minimal binding to T2 pulsed with other six peptides (Figure S2A,S2B). The other mAbs (C71‐Ab, C115‐Ab, C142‐Ab and C171‐Ab) bound to T2‐p53^R175H^ but exhibited varying levels of cross‐reactivity with the tested peptides (Figure 1H, Figure S2A,S2B).

To further validate the targeting specificity, surface plasmon resonance (SPR) analysis was conducted. The binding curves showed that C33‐Ab bound to p53^R175H^/HLA‐A*02:01 with a dissociation constant (*K*_D_) of 6.73 × 10^−8^ M but not with p53^WT^/HLA‐A*02:01 (Figure S2C). Based on the results of other four mAbs (C71‐Ab, C115‐Ab, C142‐Ab, C171‐Ab) (Figure S2D–G), C33‐Ab was identified as candidate TCRm‐Ab targeting p53^R175H^/HLA‐A*02:01, and the heavy‐ or light‐chain sequences were identified and cloned for bispecific NKCE development. An overview of the TCRm‐Ab screening process is summarized in the hourglass chart (Figure 1I). Using one HMSC‐chip, we identified a candidate TCRm‐Ab (C33‐Ab) from a half‐million hybridomas within 8 weeks.

To further investigate the cross‐reactivity of C33‐Ab, we performed an alanine scanning assay. The experimental results showed that C33‐Ab exhibited barely detectable binding to T2 cells pulsed with p53^A1^, p53^A5^ and p53^A8^ analogs, suggesting that positions 1, 5 and 8 may serve as key antigen‐binding sites for C33‐Ab (Figure S2H,S2I). In addition, through BLASTp‐based bioinformatic screening for endogenous peptides similar to HMTEVVRHC, we identified 6 similar peptides from the human canonical Swiss‐Prot proteome (UniProt ID: UP000005640) [43], and then the HLA‐A*02:01 presentation potential was predicted via NetMHCpan‐4.1 EL model (Table S2). Although the two peptides (QMSEVVQTC, QLTEVVHLC) showed comparatively strong HLA‐A*02:01 binding potential (Rank_EL < 1%), the two putative critical residues for C33‐Ab recognition at positions 1 and 8 are both mismatched with the target epitope HMTEVVRHC. These data indicate that the risk of off‐target cross‐reactivity of C33‐Ab against human self‐peptides is low. Nevertheless, we synthesized these two peptides and confirmed the result using T2 binding assay (Figure S2J).

### 2.3 Binding Ability and Specificity of scDbs

In this study, the variable region of C33‐Ab was fused with the variable region of a published anti‐CD16a (3G8) antibody to generate C33×CD16 scDb (Figures 2A,B; Table S3) [42]. A reversed version (rC33×CD16 scDb), in which the order of the two variable regions was swapped, was also synthesized for comparison (Figure 2C).

**FIGURE 2:**
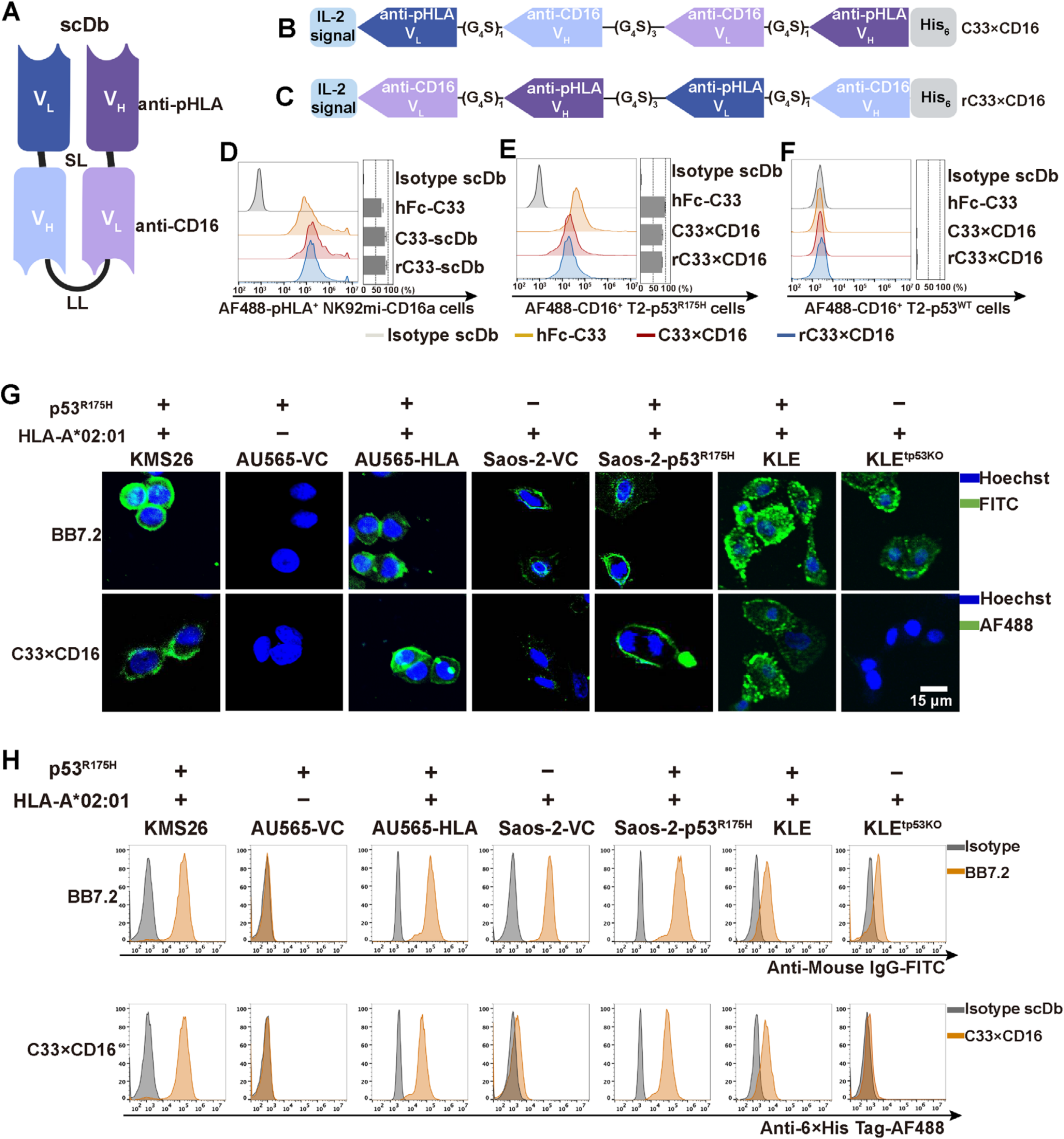
Development of C33×CD16 scDb and its cancer cell targeting assay. (A–C) Schematic of C33×CD16 and rC33×CD16 scDbs design. V_H_: heavy chain variable region; V_L_: light chain variable region; SL: short linker; LL: long linker; G: glycine; S: serine. Created in BioRender. Wu, H. (2026) https://BioRender.com/o824ht0. (D–F) FCM analysis showing binding ability of C33×CD16 and rC33×CD16 to NK92mi‐CD16a (D), T2‐p53^R175H^ (E), T2‐p53^WT^ (F). (G) Representative images of confocal fluorescence imaging that confirming C33×CD16 specifically binding to target tumor cells. (H) Representative histograms of FCM analysis that confirming specific binding of C33×CD16 to target tumor cells. AU565‐HLA: AU565 cells expressing with exogenous HLA‐A*02:01; Saos‐2‐p53^R175H^: Saos‐2 cells expressing with exogenous p53^R175H^; KLE^tp53KO^: KLE with disrupted *TP53*; “+”: positive expression; “‐”: negative expression; VC: vector control; BB7.2: HLA‐A2 antibody, Hoechst: nucleus.

We evaluated the binding properties of C33×CD16 and rC33×CD16 scDbs to the p53^R175H^/HLA‐A*02:01 complex. To verify bispecific binding, we separately tested C33×CD16 against three target cell lines: NK92mi‐CD16a, T2‐p53^R175H^, and T2‐p53^WT^. Flow cytometry (FCM) analysis showed that C33×CD16 and rC33×CD16 bound efficiently to both target cell lines (Figure 2D,E) with concentration‐dependent binding reaching approximately 80% efficiency at concentrations below 1 µg/mL (Figure S3A,S3B), but not the control T2‐p53^WT^ cells (Figure 2F).

We further assessed the cell surface retention of C33×CD16 scDb on antigen‐positive cells (NK92mi‐CD16a and T2‐p53^R175H^) using an established protocol that prevents cellular internalization, allowing evaluation of the scDb's ability to remain on the cell membrane [44]. FCM analysis showed that the C33×CD16 scDb dissociated from T2‐p53^R175H^ cells with a retention half‐life (*t*_1/2_) of 16.23 ± 1.2 min, while rC33×CD16 showed a shorter (*t*_1/2_) of 11.1 ± 0.9 min (Figure S3C). In contrast, on NK92mi‐CD16a cells, C33×CD16 displayed a retention half‐life (*t*_1/2_) of 11.65 ± 0.3 min, and rC33×CD16 showed 9.9 ± 2.1 min (Figure S3D). These results show that both C33×CD16 and rC33×CD16 exhibit stable binding to target cells.

### 2.4 C33×CD16 ScDb Maintains Binding Stability to Target Cells in Serum

The clinical potential of antibody‐based therapeutics largely depends on their serum stability [45, 46]. To evaluate this property, we measured the residual binding capacity of C33×CD16 and rC33×CD16 scDbs after incubation in human serum for defined periods using FCM. Serum half‐life (*t*_1/2_) was determined by fitting a one‐phase exponential decay curve. C33×CD16 scDb showed a *t*_1/2_ of 96.9 ± 10.2 h, while rC33×CD16 showed a shorter *t*_1/2_ of 71.4 ± 9.5 h in the T2‐p53^R175H^ cells binding model (Figure S3E). In the NK92mi‐CD16a binding model, C33×CD16 scDb had a *t*_1/2_ of 74.8 ± 5.7 h, compared to 59.2 ± 4.9 h for rC33×CD16 (Figure S3F). These results indicate that C33×CD16 and rC33×CD16 could maintain a functional activity in serum for about three days, with C33×CD16 showing superior binding affinity and stability in serum compared to rC33×CD16. Therefore, C33×CD16 was selected for further studies.

### 2.5 C33×CD16 ScDb Specifically Recognizes Cancer Cells Expressing the p53^R175H^ Mutation and HLA‐A*02:01

The C33×CD16 molecule was characterized following purification. Western blotting showed an apparent molecular weight of ∼53 kDa (Figure S3G). The purified scDb was analyzed using size exclusion chromatography (SEC). UV absorbance at 280 nm revealed a major peak eluting at 13.2 min, confirming successful purification of C33×CD16 molecule (Figure S3H). Differential scanning fluorimetry showed high thermal stability of C33×CD16, with a single melting temperature (*T*_m_) of 62.2°C (Figure S3I), indicating favorable molecular stability. SPR analysis showed that C33×CD16 bound specifically to the p53^R175H^/HLA‐A*02:01 complex with a K_D_ of 8.64 × 10^−8^ M, with no detectable binding to p53^WT^/HLA‐A*02:01 (Figure S3J,S3K).

To assess the ability of C33×CD16 scDb to recognize cancer cells, we tested it on multiple cancer cell lines with different p53 mutation and HLA‐A*02:01 expression statuses. Two endogenous cell lines (KMS26 and KLE) and two engineered cell lines (AU565‐HLA, which expresses transgenic HLA‐A*02:01; and Saos‐2‐p53^R175H^, which expresses transgenic p53^R175H^) were used as target cells. Both p53^R175H^ and HLA‐A*02:01 are expressed in all above four target cell lines. AU565‐vector control (VC) (p53^R175H^+/HLA‐A*02:01‐), Saos‐2‐VC (p53^R175H^‐/HLA‐A*02:01+) and KLE^tp53KO^ (p53^R175H^‐/HLA‐A*02:01+) (*TP53* gene disrupted by CRISPR/Cas9) served as corresponding controls. The genotyping information of HLA‐A and p53 of different cell lines was detailed in Tables S4 and S5. IF staining showed that C33×CD16 scDb specifically bound to the membrane of endogenous and engineered cells expressing both p53^R175H^ and HLA‐A*02:01 (Figure 2G), not to the control cells. FCM analysis confirmed this specific binding pattern (Figure 2H).

### 2.6 Specific Tumor Targeting of Cy5‐C33×CD16 scDb in Vivo

To assess the tumor‐targeting capability of C33×CD16 scDb, we established xenograft mouse models using three tumor cell lines: KMS26 (p53^R175H^+/HLA‐A*02:01+) as target cell line, Saos‐2 (p53^R175H^‐/HLA‐A*02:01+) and AU565 (p53^R175H^+/HLA‐A*02:01‐) as non‐target controls. Mice were administered Cy5‐conjugated C33×CD16 (Cy5‐C33×CD16) scDb or control anti‐HLA‐A2 antibody (Cy5‐BB7.2) and were monitored using an In Vivo Imaging System (IVIS) (Figure 3A). Images captured at the 6 h post‐administration showed strong fluorescent signals from Cy5‐C33×CD16 scDb specifically in KMS26 xenograft (Figure 3B), but not in Saos‐2 (p53^R175H^‐/HLA‐A*02:01+) or AU565 (p53^R175H^+/HLA‐A*02:01‐) xenografts (Figure 3B). Cy5‐BB7.2 fluorescence confirmed HLA‐A*02:01 expression in both KMS26 (p53^R175H^+/HLA‐A*02:01+) and Saos‐2 (p53^R175H^‐/HLA‐A*02:01+) xenografts (Figure 3B). Fluorescent signals of Cy5‐C33×CD16 scDb in KMS26 xenografts peaked around 6 h post‐administration (Figure S4A) and rapidly declined, becoming undetectable after 96 h (Figure S4B), indicating fast pharmacokinetic clearance of Cy5‐C33×CD16 scDb. Ex vivo imaging at 6 h post‐administration showed that Cy5‐C33×CD16 was accumulated significantly only in KMS26 xenograft, not in Saos‐2 and AU565 xenografts and all other organs (heart, liver, spleen, lung, and kidney) derived from three tested xenograft models (Figure 3C–I). FCM analysis of xenograft‐derived single‐cell suspensions confirmed Cy5‐C33×CD16 accumulation specifically in KMS26 xenografts (Figure S4C,S4D). These results provide preliminary evidence of tumor‐selective targeting and promising in vivo circulation behavior in mice, supporting its potential for precision therapy of p53^R175H^/HLA‐A*02:01‐positive cancers.

**FIGURE 3:**
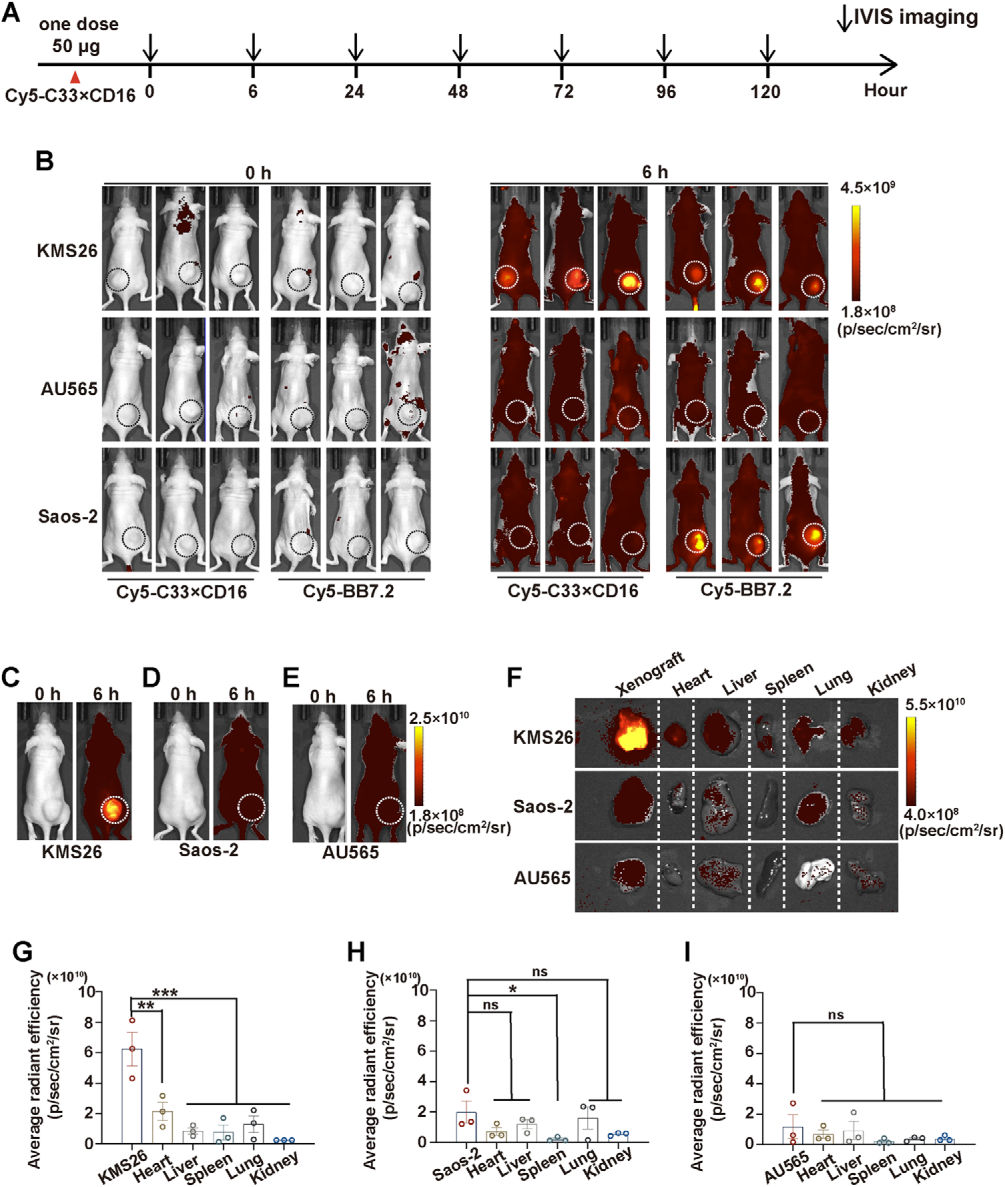
Tumor‐specific targeting of C33×CD16 in vivo. (A) Experimental workflow of in vivo targeting evaluation. Three nude mouse xenograft models (KMS26, Saos‐2, AU565) were injected Cy5‐C33×CD16 or Cy5‐BB7.2. Images were captured at indicated timepoints using IVIS. (B) Images showing the signal distribution of Cy5‐C33×CD16 or Cy5‐BB7.2 in three xenograft models at 0‐h and 6‐h timepoint. Dashed circles indicate tumor locations. (C–E) Parallel experiment evaluating Cy5‐C33×CD16 biodistribution at 6‐h timepoint. Images show the signal distribution of Cy5‐C33×CD16 in three xenograft models, KMS26 (C), Saos‐2 (D), AU565 (E). (F) Representative images of tumor and five organs showing biodistribution. (G–I) Quantitative analysis of average radiant efficiency in xenografts and five organs from xenograft models, KMS26 (G), Saos‐2 (H), AU565 (I). Data are presented as means ± s.d. (n = 3 biologically independent mice). Statistical significance was determined by one‐way ANOVA followed by Dunnett's multiple comparisons test (all groups vs. KMS26), or by Kruskal–Wallis test followed by Dunn's multiple comparisons test (all groups vs. Saos‐2, all groups vs. AU565). “*”: *p* < 0.05, “**”: *p* < 0.01, “***”: *p* < 0.001, ns: not significant, *p* > 0.05.

### 2.7 C33×CD16 ScDb Enhances NK Cell‐mediated Cytotoxicity Against p53^R175H^/HLA‐A*02:01 Cancer Cells

Antibody‐dependent cellular cytotoxicity (ADCC) is a key immune mechanism by which antibodies recruit cytotoxic immune cells (e.g., NK cells) to specifically destroy target tumor cells [47, 48]. We hypothesized that C33×CD16 scDb, functioning as a bispecific NKCE, could strengthen the interaction between target cancer cells and NK cells, and activate NK cells via anti‐CD16a (3G8) antibody, thereby enhancing antitumor activity (Figure 4A) [49]. To evaluate this hypothesis, we co‐cultured NK92mi‐CD16a cells with various target cancer cells (KMS26, KLE, AU565‐HLA, Saos‐2‐p53^R175H^) that express both p53^R175H^ and HLA‐A*02:01, as well as control cells (Saos‐2‐VC, AU565‐VC) that lack either p53^R175H^ or HLA‐A*02:01. The supernatants were tested using enzyme‐linked immunosorbent assay (ELISA), which demonstrated that C33×CD16 specifically induced IFN‐γ secretion by NK92mi‐CD16a cells in response to target cells, but not to control cells, suggesting its capacity to activate NK cell‐mediated cytotoxicity when bridging NK cells and target cancer cells (Figure 4B). Further, cell death was assessed using FCM by measuring propidium iodide (PI) incorporation in carboxyfluorescein succinimidyl ester (CFSE)‐labeled target cells (Figure S5A). The results revealed dose‐dependent cytotoxicity in all tested target cells treated with hFc‐C33 and C33×CD16 scDb, but not in control cells (Figure 4C–J). We also found that C33×CD16 induced a concentration‐dependent increase in the levels of these cytotoxic markers (IFN‐γ, TNF‐α, Perforin, Granzyme‐B, IL‐2) in KMS26 co‐culture model, further supporting its ability to elicit NK cell‐mediated cytotoxicity (Figure S5B). Similar results were observed in Calcein‐AM (green) and PI (red) double staining assays (Figure S5C). Notably, C33×CD16 induced significantly stronger NK cell‐mediated cytotoxicity than hFc‐C33 did across all tested target cells, confirming its superior therapeutic potential (Figure S5D). Further, primary NK cells isolated from peripheral blood mononuclear cells (PBMCs) of a healthy donor can also effectively kill target tumor cells without killing control cells (Figure S5E–G). Collectively, these findings demonstrate that C33×CD16 scDb specifically and effectively triggers NK‐mediated cytotoxicity against p53^R175H^/HLA‐A*02:01‐expressing tumor cells.

**FIGURE 4:**
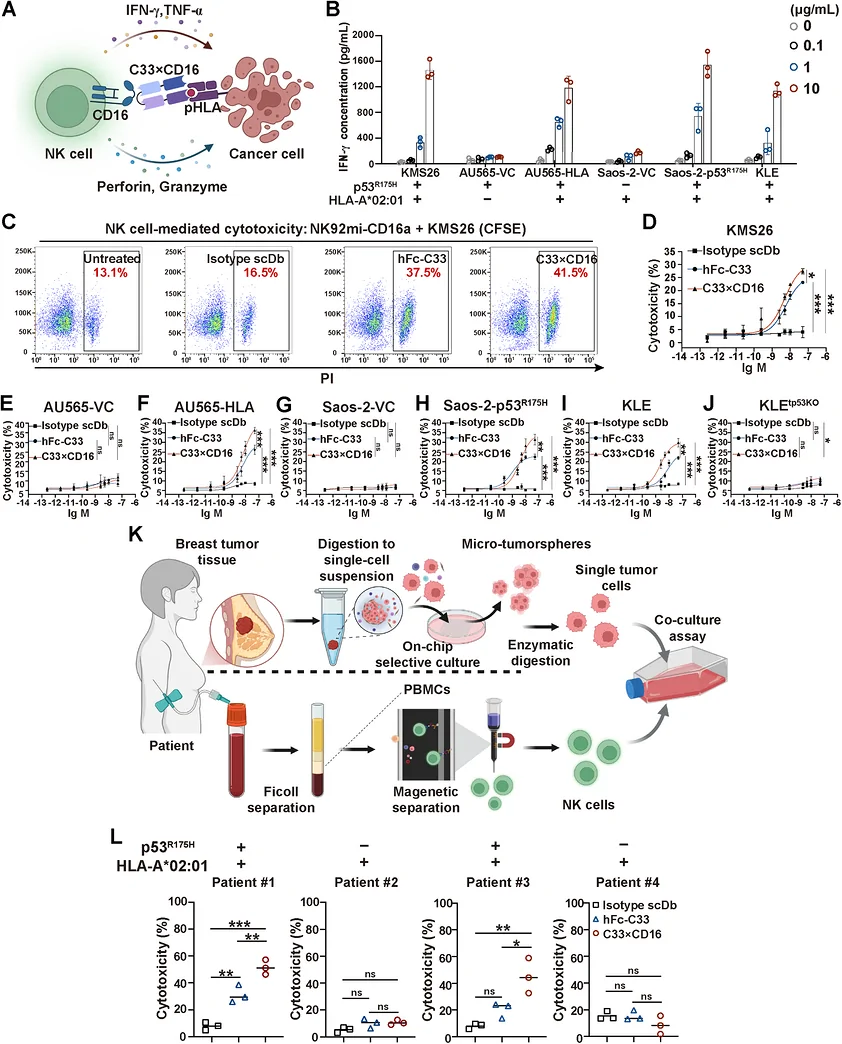
Enhanced NK cell‐mediated cytotoxicity by C33×CD16. (A) Schematic illustrating how C33×CD16 enhances NK cell‐mediated cancer cell killing. Created in BioRender. Wu, H. (2026) https://BioRender.com/o824ht0. (B) Initial assessment of NK cell‐mediated cytotoxicity induced by different concentrations of C33×CD16 (0.1 µg/mL, 1 µg/mL, 10 µg/mL), measured by ELISA for IFN‐γ secretion (E:T ratio at 1:1). (C) Representative dot plots show the percentage of PI‐positive KMS26 cells. (D–J) Various tumor cell lines with different HLA expression and p53^R175H^ status were co‐cultured with NK92mi‐CD16a cells at an E:T ratio of 10:1 to evaluate NK cell‐mediated cytotoxicity. Cells were treated with isotype, hFc‐C33 or C33×CD16, or left untreated. All data were normalized by subtracting the data of the untreated group and presented as a line graph, showing results for different tumor cell lines KMS26 (D), AU565‐VC (E), AU565‐HLA (F), Saos‐2‐VC (G), Saos‐2‐p53^R175H^ (H), KLE (I), KLE^tp53KO^ (J). (K) Schematic illustration for the co‐culture assay using patient‐derived micro‐tumorspheres (MTSs) cells and primary NK cells. Created in BioRender. Wu, H. (2026) https://BioRender.com/3povypo. (L) Statistical results showing the cytotoxicity elicited by primary NK cells toward tumor cells derived from MTSs of Patient #1, #2, #3 and #4 in the presence of C33×CD16. AU565‐HLA: AU565 cells expressing with exogenous HLA‐A*02:01; Saos‐2‐p53^R175H^: Saos‐2 cells expressing with exogenous p53^R175H^ peptides; “+”: positive expression; “‐”: negative expression; VC: vector control. Data represent mean ± s.d.; *n* = 3 biological replicates. Statistical significance was determined using one‐way ANOVA followed by Tukey's multiple comparisons test. “*”: p < 0.05, “**”: p < 0.01, “***”: p < 0.001, ns: not significant, p > 0.05.

To assess whether the C33×CD16 scDb can trigger NK cell‐mediated cytotoxicity against patient‐derived tumor cells that naturally express both the p53^R175H^ mutation and HLA‐A*02:01, we generated micro‐tumorspheres (MTSs) through selective culture on hydrogel‐based microwell cell‐chip (HMWC‐chip) (Figure S6A,S6B,S6C–F), and co‐cultured MTS‐derived tumor cells with autologous primary NK cells isolated from the same patients for cytotoxicity assays (Figure 4K). The HLA‐A*02:01 expression and p53^R175H^/HLA‐A*02:01 presentation were confirmed using flow cytometry (Figure S6G). The clinical and molecular information of patients was detailed in Table S6. In Patient #1 and #3 expressing both p53^R175H^ and HLA‐A*02:01, C33×CD16 induced robust, antigen‐specific NK cell cytotoxicity, significantly exceeding the activity of the control hFc‐C33 (Figure 4L). In contrast, in Patient #2 and #4 expressing wild‐type p53 (p53^WT^) and HLA‐A*02:01, no significant cytotoxicity was observed upon C33×CD16 treatment (Figure 4L). These results suggest that C33×CD16 mediates target‐dependent NK cell cytotoxicity against tumor cells endogenously expressing p53^R175H^/HLA‐A*02:01 at physiologically levels, supporting its clinical translation potential.

In addition, the full 3G8 antibody activates CD16b on neutrophils only when its Fc domain engages Fc gamma RIIa and the Fab bind to two Fc gamma RIIIb (CD16b) molecules [50]. In contrast, the C33×CD16, composed of dual scFvs, lacks this dual‐engagement capability and therefore cannot activate neutrophils, thus minimizing CRS risk. To directly assess CRS potential, we treated human PBMCs with C33×CD16 and measured IL‐8, IL‐6, TNF‐α, and IFN‐γ levels by ELISA. As expected, C33×CD16 caused only a slight TNF‐α increase and no significant change in IL‐6 or IL‐8; all cytokine levels remained far below those induced by the lipopolysaccharide (LPS) and TGN1412 (positive controls) (Figure S7). These data confirm that C33×CD16 drives target‐dependent NK cell activation without systemic cytokine release or CRS.

### 2.8 In Vivo Therapeutic Efficacy of C33×CD16 scDb

We further evaluated its therapeutic potential in vivo in a xenograft NSG mice model (KLE, p53^R175H^+/HLA‐A*02:01+) (Figure 5A). Survival rate demonstrated that mice treated with C33×CD16 or hFc‐C33 exhibited significantly prolonged survival compared to the isotype control (Figure 5B). By Day 33, the average tumor volumes in both C33×CD16 scDb‐treated, and hFc‐C33‐treated mice remained below 400 mm^3^, whereas those in the control groups (isotype) is about 700 mm^3^ (Figure 5C). All control animals reached the ethical endpoint (tumor volume > 1500 mm^3^) or died before Day 50 (Figure 5D), while both C33×CD16 and hFc‐C33 treatments exhibited clear antitumor effects (Figure 5D). Notably, C33×CD16 exhibited superior efficacy, with four animals surviving beyond 2 months (Figure 5D). To assess whether C33×CD16‐ or hFc‐C33‐induced NK‐mediated cytotoxicity contributed to tumor inhibition, tumor tissues were digested to single‐cell suspension for FCM analysis (Figure 5E–G). The results revealed significantly higher NK cell infiltration in xenografts treated with C33×CD16 than with hFc‐C33 (Figure 5E,F), along with approximately tenfold increase in the absolute counts of IFN‐*γ*‐secreting NK cells (Figure 5G). These results support the therapeutic potential of C33×CD16 scDb.

**FIGURE 5:**
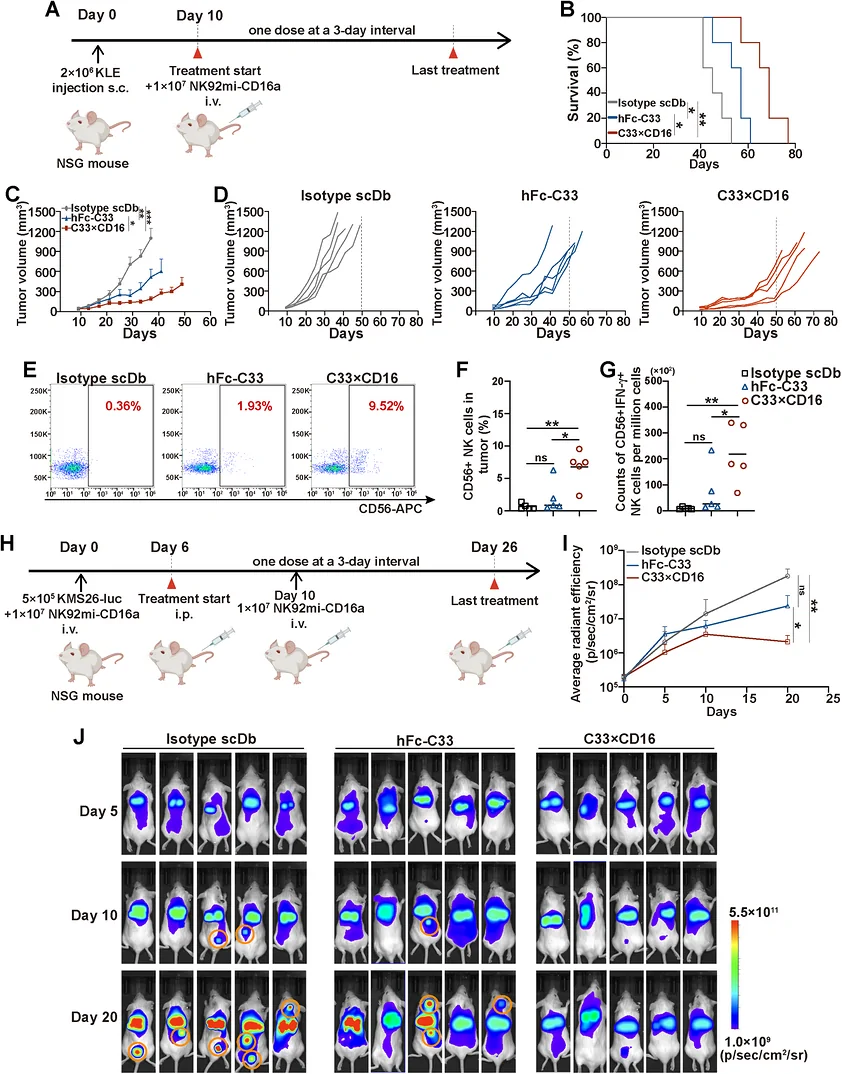
Antitumor efficacy of C33×CD16 in vivo. (A–G) Evaluation of antitumor efficacy of C33×CD16. Experimental timeline for animal study (A). KLE xenograft models were established using NSG mice. Mice were intravenously (IV) administered hFc‐C33, C33×CD16, isotype control every 3 days. Survival rate curves for each treatment group, analyzed by the Kaplan–Meier method (B). Statistical significance was determined using the log‐rank test. “*”: *p* < 0.05, “**”: *p* < 0.01. Average tumor volume in mice treated with isotype (gray), hFc‐C33 (blue), or C33×CD16 (red) until first death (C). Data represent mean ± S.D.; *n* = 5 biologically independent mice per group. Tumor growth curves of individual mouse (D). Statistical significance of data on day 37 was determined using one‐way ANOVA followed by Tukey's multiple comparisons test. “*”: *p* < 0.05, “**”: *p* < 0.01, “***”: *p* < 0.001. Representative dot plots show the percentage of infiltrating NK92mi‐CD16a cells (CD56^+^) in tumor tissue (E). Statistical result shows the percentage of infiltrating NK92mi‐CD16a cells (CD56^+^) in tumor tissue (F). Statistical result shows the counts of infiltrating NK92mi‐CD16a cells (CD56^+^) secreting IFN‐γ per million cells (G). Data represent mean ± s.d.; *n* = 5 biologically independent mice per group. Statistical significance determined using Kruskal–Wallis test followed by Dunn's multiple comparisons test. “*”: *p* < 0.05, “**”: *p* < 0.01, ns: not significant, *p* > 0.05. (H–J) Evaluation of antitumor efficacy of C33×CD16 combined with adoptive NK cell transfer. Experimental timeline (H). Xenograft models were established by IV injection of KMS26‐luc cells into NSG mice. Mice were IV administered hFc‐C33, C33×CD16, isotype control every 3 days. (I) Average tumor volume in mice treated with isotype (gray), hFc‐C33 (blue), or C33×CD16 (red) until day 20. (J) IVIS imaging of individual mice on days 5, 10, 20 post‐treatments. Orange circle: location of metastatic lesion. Data represent mean ± s.d.; *n* = 5 biologically independent mice per group. Statistical significance of data on day 20 was determined using Kruskal–Wallis test followed by Dunn's multiple comparisons test. “*”: *p* < 0.05, “**”: *p* < 0.01, ns: not significant, *p* > 0.05. Partially created in BioRender. Wu, H. (2026) https://BioRender.com/7z2oryq.

To further evaluate the therapeutic potential of C33×CD16 scDb in combination with NK cell therapy, we established a xenograft model in NSG mice by intravenous (IV) injection of KMS26‐luc (5 × 10^5^ cells) and NK92mi‐CD16a (1 × 10^7^ cells) (Figure 5H). In vivo imaging demonstrated that hFc‐C33 moderately inhibited tumor growth by Day 20 (Figure 5I). C33×CD16‐treated mice exhibited significantly lower fluorescence intensity than hFc‐C33‐treated mice did, indicating more effective tumor suppression of C33×CD16 treatment. Notably, C33×CD16‐treated mice showed almost no metastatic signals, confirming that C33×CD16 potently suppresses metastatic lesion formation (Figure 5J). These results indicate that C33×CD16 scDb not only demonstrates potent antitumor efficacy in vivo but also holds promise for synergizing adoptive NK cell transfer immunotherapy.

### 2.9 In Vivo Safety Evaluation of C33×CD16 scDb

To address concerns regarding potential off‐target binding of TCRm‐Ab to the widely distributed HLA molecule on nucleated cell membranes, we assessed the in vivo toxicity of C33×CD16 scDb and hFc‐C33 in MHC‐humanized mice (NCG‐HLA‐A2.1) with IV injection of NK92mi‐CD16a cells (Figure 6A). C33×CD16‐ and hFc‐C33‐treated mice showed no significant differences in body weight compared to phosphate‐buffered saline (PBS)‐treated controls, indicating no adverse effects on general health (Figure 6B,C). Furthermore, histological examination by hematoxylin and eosin (H&E) staining of five major organs, including heart, liver, spleen, lungs, and kidney, showed no detectable differences among C33×CD16‐, hFc‐C33‐, and PBS‐treated mice (Figure 6D), indicating no tissue damage in HLA‐A2‐expressing organs. Collectively, these findings confirm that no obvious adverse toxic responses were observed in mice under the tested experimental conditions, supporting the translational potential of C33×CD16 for clinical applications.

**FIGURE 6:**
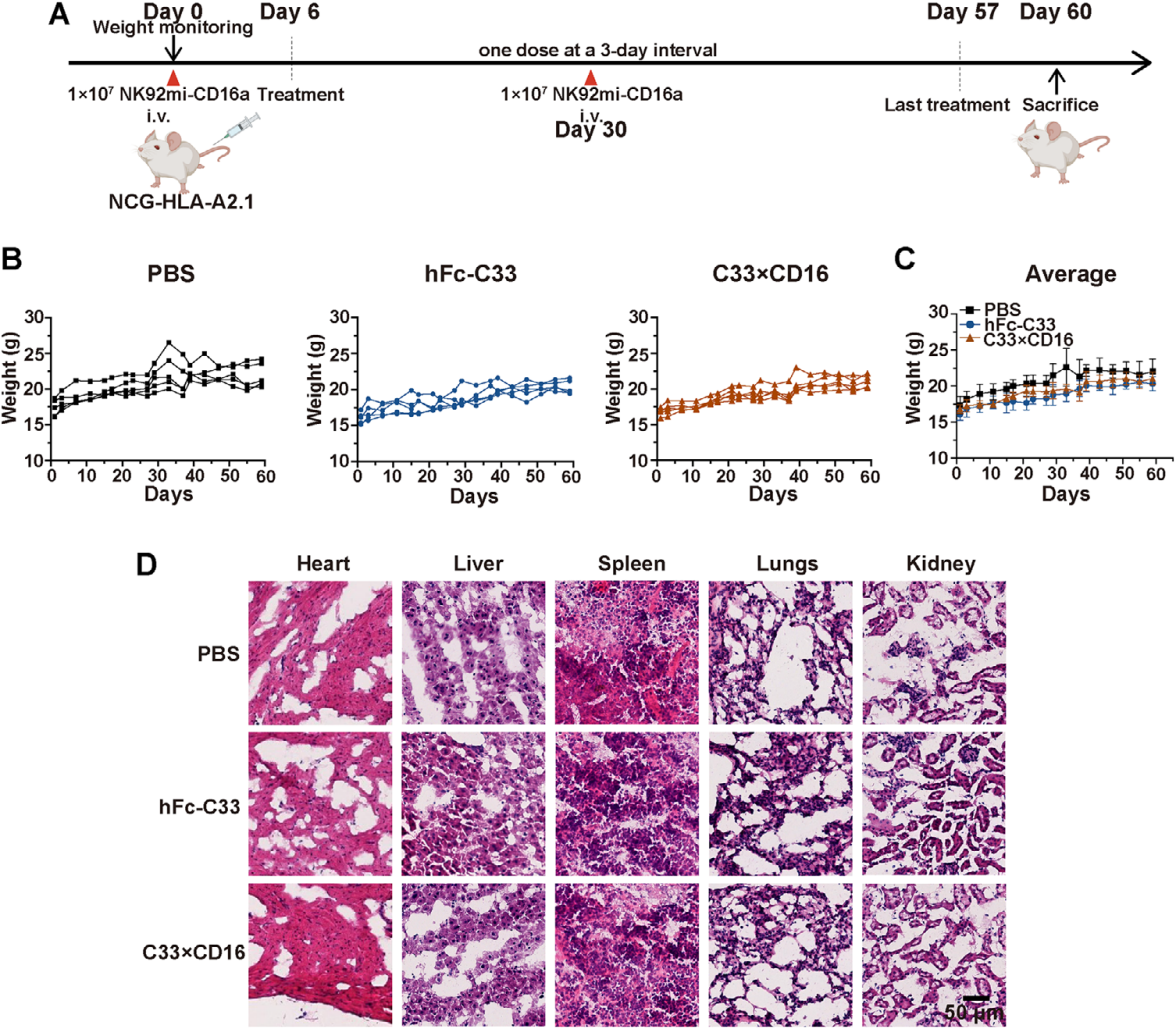
In vivo safety evaluation of C33×CD16 treatment. (A) Experimental timeline. MHC humanized mice (NCG‐HLA‐A2.1) were intraperitoneally (IP) administered C33×CD16 (2 mg/kg/dose) or hFc‐C33 (6 mg/kg/dose), or PBS as a vehicle control every 3 days. NK92mi‐CD16a (1×10^7^ cells/dose) was injected on day 0, 30, respectively (one dose each day). (B) Individual body weight curves for PBS, hFc‐C33, C33×CD16 treatments. (C) Average body weight in mice treated with PBS (gray), hFc‐C33 (blue), C33×CD16 (red). (D) Histological examination by H&E staining of five organs (heart, liver, spleen, lungs, and kidney) from mice treated with hFc‐C33, C33×CD16 and PBS control. Partially created in BioRender. Wu, H. (2026) https://BioRender.com/7z2oryq.

## 3 Discussion

Our study presents three key results. First, we developed a single hybridoma‐based method for TCRm‐Ab discovery by integrating high‐throughput screening of single hybridomas on HMSC‐chip with de‐selection based on T2 cell binding assay. Using this approach, we identified C33‐Ab that specifically recognizes the p53^R175H^ neoantigen presented by HLA‐A*02:01. Second, we designed a novel bispecific NKCE, C33×CD16, a scDb composed of one scFv arm derived from C33‐Ab, and the other scFv arm derived from the CD16‐specific antibody 3G8. Third, C33×CD16 significantly enhances tumor‐specific NK cell‐mediated cytotoxicity and exhibits strong synergistic therapeutic efficacy when combined with adoptive NK cell transfer, highlighting its potential for precision cancer immunotherapy.

Neoantigen‐derived TCRm‐Abs have recently been applied to develop BiTEs [24, 51], showing promise in precision oncology by mitigating the off‐target toxicity commonly associated with TAA‐based BiTEs. However, the application of neoantigen‐targeting TCRm‐Abs in bispecific NKCE design remains unexplored. Some tumors evade NK cell surveillance by maintaining or upregulating MHC‐I expression, which engages NK cell inhibitory receptors such as KIRs and NKG2A [15, 16]. Notably, because neoantigens are presented by MHC‐I, elevated MHC‐I expression promotes the surface presentation of neoantigen‐derived peptide‐MHC‐I (pMHC‐I) complexes, thereby increasing the target availability for TCRm‐Abs. This logically augments the efficacy of TCRm‐Ab‐based bispecific NKCEs in NK cell‐mediated immunotherapy. Therefore, the NK cell‐mediated cytotoxicity triggered by TCRm‐Ab‐based bispecific NKCEs may to some extent weaken the immunosuppressive effect caused by MHC‐I–mediated NK cell immune evasion in the tumors.

TCRm‐Abs specifically recognize neoantigens in an HLA‐restricted manner, leading to their extreme rarity in antibody repertoire derived from animals immunized with pHLA complexes. This scarcity presents a major challenge in TCRm‐Ab screening. Traditional methods, such as hybridoma limiting dilution and phage display, are limited by low throughput or poor precision, making them both time‐consuming and labor‐intensive [22, 52, 53, 54, 55, 56, 57, 58, 59, 60].

Advanced microwell chip‐based technologies allow high‐throughput screening of antigen‐specific antibodies from single antibody‐secreting cells (ASCs) by analyzing in situ antibody secretion at the single‐cell level [61, 62, 63]. The recently emerged Beacon Optofluidic System (Berkeley Lights, Inc.) enables antibody secretion monitoring from >10,000 ASCs per cell chip for selection of antigen‐specific antibodies [64]. However, its throughput is insufficient for isolating extremely rare TCRm‐Abs. In this study, we established a robust TCRm‐Ab discovery method based on high‐throughput screening of single hybridomas on HMSC‐chip (> 500,000 cells per chip), followed by de‐selection based on T2 cell binding assay. This method fulfills the critical requirements for TCRm‐Ab discovery at the single cell level: comprehensive high‐throughput coverage of ASCs and effective de‐selection using control antigens. Our results prove that HMSC‐chip enables high‐throughput, rapid, and precise discovery of functional TCRm‐Abs at the single‐cell level, thereby accelerating the development of TCRm‐Ab‐based therapeutics. Nevertheless, this strategy exhibits relatively low efficiency for identifying optimal TCRm antibodies from a large pool of positive monoclonal clones (1/268). This leads to considerable downstream validation work. We are currently designing a direct de‐selection step on HMSC‐chip to further improve screening precision and overall efficiency.

As a lab‐developed bispecific NKCE, the C33×CD16 is not yet a viable therapeutic candidate at the present stage. It requires rigorous optimization and validation, including humanization, specificity profiling, efficacy verification, and safety evaluation, before advancing to clinical trials.

The future optimization strategy for our C33×CD16 will primarily focus on sequence humanization. The anti‐CD16 arm derived from 3G8 can be humanized using well‐established CDR‐grafting frameworks with validated clinical applicability [65]. Notably, the 3G8‐based construct in this study was only used for proof‐of‐concept validation. For the C33 TCRm arm, structure‐guided CDR grafting onto matched human germline frameworks [66, 67], combined with targeted framework back‐mutations at Vernier zone residues, to avoid affinity loss during CDR grafting [68]. Additional strategies, including specificity‐determining residue (SDR) grafting, chain shuffling and artificial intelligence (AI)‐assisted sequence design [69, 70], as well as the generation of fully human TCRm antibodies via human transgenic mouse immunization [71, 72], will further advance candidate maturation.

Notably, slight but significant (*p* = 0.032) cytotoxicity was still observed against *TP53*‐knockout KLE cells expressing both HLA‐A*02:01 and HLA‐A*24:02, which is consistent with the weak binding signal of KLE^tp53KO^ cells shown in Figure 2h. This suggests weak off‐target recognition, potentially toward alternative HLA allotypes such as HLA‐A*24:02, which requires further validation. Such promiscuous HLA recognition may broaden patient applicability but also introduces risks of on‐target/off‐tumor toxicity, highlighting the necessity of high‐throughput peptide screening and systematic preclinical safety assessment for TCRm‐based therapeutics [73, 74]. This balance between expanded therapeutic utility and on‐target, off‐tumor toxicity demands rigorous preclinical specificity profiling, including high‐throughput peptide library screening, prior to clinical translation. Our current off‐target screening is predicated on sequence homology alignment and alanine scanning‐defined key recognition residues. A more comprehensive strategy, including full positional substitution scanning and proteome‐wide motif‐based cross‐reactivity prediction across all HLA‐A*02:01‐restricted peptides, will be implemented in future studies to comprehensively define the TCRm cross‐reactivity landscape.

Current C33×CD16 construct with a scDb format facilitates tumor penetration but results in a short systemic circulation half‐life, which may hinder sustained in vivo efficacy and complicate clinical administration. Multiple engineering strategies can be applied to optimize pharmacokinetic properties. Fc fusion with effector‐silenced mutations (LALA or N297Q) can extend circulation time via FcRn recycling, though its trade‐off between enlarged molecular size, tumor penetration capacity, and residual Fc‐mediated immune activation requires careful evaluation [75, 76]. Alternatively, albumin‐binding domain conjugation enables half‐life extension with minimal size increment, better preserving tumor‐homing performance [77]. Site‐specific PEGylation and IgG‐like bispecific reformatting can also improve stability and manufacturability [78, 79], while their impacts on synapse formation, tumor penetration, and therapeutic functionality need systematic characterization. Overall, rational molecular optimization balancing tumor delivery, NK activation potency, and clinical safety will advance the translational potential of TCRm‐NKCEs for targeting intracellular oncogenic mutations.

In summary, this work presents a robust method for the rapid and precise screening of rare TCRm‐Abs and for design of TCRm‐Ab‐based bispecific NKCEs. This is the first report of an engineered TCRm‐Ab‐based bispecific NKCE incorporating 3G8 that has been successfully employed in the development of bispecific antibodies [42, 80, 81] for NK cell‐mediated immunotherapy. This proof‐of‐concept work demonstrates that it is feasible to generate bispecific NKCEs based on neoantigen‐targeting TCRm‐Abs that can specifically recognize neoantigens, such as p53^R175H^. This study provides a promising strategy to exquisitely enhance tumor‐specific NK cell‐mediated immunotherapy by targeting intracellular oncogenic hotspot mutations. Notably, additional validation with more patient‐derived micro‐tumorspheres is needed to support the clinical translational value of this finding. Also, safety testing with PBMCs from one healthy donor showed no CRS risk for C33×CD16, further assessments with expanded healthy‐donor samples are planned to account for individual differences. Further investigations are required to optimize the design of the TCRm‐Ab‐based bispecific NKCE (C33×CD16 scDb) to minimize potential side effects and maximize therapeutic efficacy, and to assess its therapeutic potential using patient‐derived organoids, with the goal of advancing into clinical trials.

## 4 Materials and Methods

### 4.1 Peptide and pHLA Complexes

The peptides utilized in this study, including p53^R175H^, p53^WT175^, p53^A5^, p53^A1^, Survivin, Flu, and WT‐1, were chemically synthesized by GenScript (Nanjing, China) with a purity of ≥ 95%. The specific sequences are provided in Table S1. Stock solutions (5 mg/mL) were prepared in dimethylformamide (DMF) and stored at ‐20°C. Human β2‐microglobulin (β2M) (Prospec Bio, East Brunswick, NJ, USA) was reconstituted in phosphate buffered saline (PBS) at 10 mg/mL and stored at ‐20°C. The pHLA complexes p53^R175H^/HLA‐A*02:01 and p53^WT175^/HLA‐A*02:01 were purchased from Novoprotein Scientific Inc. (Suzhou, China).

### 4.2 High‐throughput TCRm‐Ab Screening Based on HMSC‐chip Technology

The p53^R175H^/HLA‐A*02:01 pHLA were labeled with Alexa Fluor 488 (green) fluorescence using Alexa Fluor Succinimidyl Esters (Thermofisher scientific, MA, USA) following the manufacturer's instructions. The labeled pHLA was added into RPMI‐1640 medium at a final concentration of 10 µg/mL in a 6‐cm plate containing a HMSC‐chip loaded with p53^R175H^/HLA‐hybridomas. The hybridomas were cultured for 3 h in a CO_2_ incubator. Following incubation, the HMSC‐chips were scanned and imaged using CytoStar equipment (LivingChip, Nanjing, China). Fluorescence rings (FRs) surrounding hybridomas on the HMSC‐chip were precisely analyzed; selected single hybridomas were automatically transferred into individual wells of a 96‐well plate for further clonal expansion and antibody production.

### 4.3 T2 Cell Binding Assay and Alanine Scanning Assay

Transporters associated with antigen processing (TAP)‐deficient T2 cells (HLA‐A*02:01‐expressing) were cultured in RPMI‐1640 medium supplemented with 10% fetal bovine serum (FBS), 2 mM L‐glutamine, and 1% penicillin‐streptomycin (P/S) at 37°C in a humidified incubator with 5% CO_2_. To evaluate the binding efficiency of antibodies from hybridoma supernatants, T2 cells were incubated overnight in fresh medium containing 10 µg/mL human β_2_‐microglobulin (β_2_M) (PRO‐337, ProSpec, USA) and 5 µg/mL of antigenic peptides (p53^R175H^ or p53^WT^) to allow the formation of pHLA complexes on the T2 cell surface (T2‐p53^R175H^ as target, T2‐ p53^WT^ as control). T2 cells displaying pHLA complexes were seeded at 1 × 10^5^ cells per well in a 96‐well plate and incubated with hybridoma supernatants (final IgG concentration: 0.5 µg/mL) for 2 h at 4°C. After two washes, the cells were stained with FITC‐labeled Goat Anti‐Mouse IgG (H+L) (A0568, Beyotime, Shanghai, China) for 2 h at 4°C, followed by flow cytometry (FCM) analysis. For alanine scanning assay, a series of peptides with single amino acid substitutions to alanine (p53^A1^ to p53^A9^) were generated and used for T2 binding assay as described above. The peptide sequences used are listed in Table S1.

### 4.4 IF Analysis of scDb Binding to Cancer Cells

To evaluate the binding of C33×CD16 scDb to p53^R175H^/HLA‐A*02:01 pHLA complexes on cell surfaces, IF staining was performed on various cancer cell lines (KMS26, KLE, AU565‐VC, Saos‐2‐VC, AU565‐HLA, Saos‐2‐p53^R175H^ and KLE^tp53KO^). Cells were seeded in 3.5 cm culture dishes and incubated overnight. Then cells were fixed with 4% paraformaldehyde for 10 min at room temperature. After blocking with PBS containing 1% bovine serum albumin (BSA) for 1 h, cells were washed with PBS. C33×CD16 scDb or control anti‐HLA‐A2 (BB7.2) antibody (343302, BioLegend) were added (10 µg/mL in PBS) and incubated at 4°C for 2 h. Following three washes, the cells were incubated with either 6×His Tag Monoclonal Antibody conjugated to AF488 (MA1‐21315‐A488, ThermoFisher Scientific, MA, USA) or FITC‐labeled Goat Anti‐Mouse IgG (H+L) (A0568, Beyotime, Shanghai, China), both diluted 1:500 in PBS, for 2 h at 4°C. After three washes with PBS, fluorescent images were captured using an Olympus FV3000 confocal laser scanning microscope.

### 4.5 Evaluation of scDb‐dependent Cytotoxicity Elicited by NK92mi‐CD16a

Target tumor cell lines (KMS26, Saos‐2‐VC, Saos‐2‐p53^R175H^, AU565‐VC, AU565‐HLA, KLE and KLE^tp53KO^) were adjusted to 1 × 10^6^ cells/mL. The cells were labeled using carboxyfluorescein succinimidyl ester (CFSE) (C0051, Beyotime, Shanghai, China) according to the manufacturer's instruction. Then, 100 µL of the target cell suspension was seeded into U‐bottom 96‐well plates, and effector cells (NK92mi‐CD16a) were added to the wells at an effector‐to‐target (E:T) ratio of 10:1 in the presence of C33×CD16 scDb (2.5 × 10^−13^ M to 5 × 10^−8^ M). The co‐cultures were incubated for 24 h at 37°C in a 5% CO_2_ incubator. After centrifugation at 200 g for 5 min, the supernatants were collected for cytokine quantification. The harvested cells were rinsed three times with PBS and then suspended in 200 µL of PBS containing 1% FBS. Propidium iodide (PI) (ST511, Beyotime, Shanghai, China) was added to the cell suspension at a final concentration of 5 µM. The cells were assessed by FCM using a CytoFLEX LX instrument (Beckman Coulter) to quantify the proportion of dead cancer cells.

### 4.6 Generation of Micro‐tumorspheres (MTSs) Derived from Primary Breast Cancer Tumor Tissues

MTSs are three‐dimensional spherical structures generated by the clonal proliferation of tumor‐initiating cells, which are commonly characterized as cancer stem cells [82, 83, 84]. MTSs were generated using our currently published method [85]. To produce MTSs from clinical breast cancer tumor samples, surgical specimens were minced and digested in 5 mL DMEM/F12 culture medium containing 1 mg/mL Collagenase Type I (SCR103, Sigma‐Aldrich, Shanghai, China), 0.5 mg/mL Hyaluronidase (HX0514‐1, Sigma‐Aldrich, Shanghai, China), 50 µg/mL DNase I (10104159001, Sigma‐Aldrich, Shanghai, China) (digestion enzyme mix) for 90 min at 37°C with gentle inversion every 10 min. Digestion was halted with 5 mL DMEM/F12 with 10% FBS, and the suspension was filtered twice through a 45‐µm strainer. Subsequently, cells were centrifuged then resuspended in 15 mM HEPES‐supplemented F‐12 SM (36254, STEMCELL Technologies) and seeded onto hydrogel‐based microwell cell‐chip (HMWC‐chip) with 80 µm diameter of microwells. After 3 days of culture in plain F‐12 SM, the medium was replaced with MammoCult Human Medium (05620, STEMCELL Technologies) containing 0.5 µg/mL hydrocortisone (07925, STEMCELL Technologies) and 4 µg/mL heparin (07980, STEMCELL Technologies), followed by 4 more days of incubation at 37°C and 5% CO_2_ to support MTSs formation. MTSs (about 60 µm diameter) formed in microwells were collected using a hydrodynamics actuated cell sorting (HACS) technology for co‐culture assays [86].

### 4.7 Co‐Culture Assay of Primary Human NK Cell Cytotoxicity With Cell Lines or Patient‐Derived MTSs

The peripheral blood mononuclear cells (PBMCs) were isolated using Ficoll Plus 1.077 (P4350, Solarbio, Beijing, China) following the standard protocol. The human primary NK cells were isolated from PBMCs using NK Cell Isolation Kit (130‐092‐657, Miltenyi Biotec Inc., CA, USA) following the instruction of manufacturer.

To evaluate the primary human NK cell cytotoxicity towards tumor cell lines, a co‐culture assay was conducted following a similar method described above (Evaluation of scDb‐dependent cytotoxicity elicited by NK92mi‐CD16a).

To assess cytotoxicity of primary human NK cells against autologous breast cancer cells under C33×CD16 scDb treatment, we co‐cultured MTS‐derived tumor cells with matched NK cells. Briefly, MTSs generated from patient‐derived tumor tissues on HMWC‐chips were collected, washed twice with 1 mL PBS, and dissociated with 0.25% trypsin (500 µL) for 3 min at 37°C. The enzymatic reaction was terminated with 2 mL of DMEM/F12 containing 10% FBS. MTS‐derived tumor cells were then centrifuged at 300 g for 5 min. The resulting tumor cells were used as target cells and autologous primary NK cells served as effector cells following the same co‐culture method described above. The HLA‐A*02:01 expression and p53^R175H^/HLA‐A*02:01 presentation were confirmed using the anti‐HLA‐A2 BB7.2 antibody (343302, BioLegend) and p53^R175H^/HLA‐A*02:01‐targeting antibody H2 (ARO‐A17387‐50, ProteoGenix). The single cells from patient‐derived MTSs were incubated with the anti‐p53^R175H^/HLA‐A*02:01 primary antibody H2, following FITC‐labeled goat anti‐mouse IgG (H+L) (A0568, Beyotime) secondary antibody staining. After washing, the cells were analyzed using flow cytometry.

### 4.8 In Vivo Assessment of C33×CD16 scDb Tumor Targeting

To evaluate the tumor‐specific binding capacity of C33×CD16 scDb in vivo, seven‐week‐old nude mice were subcutaneously (SC) injected with 1 × 10^6^ tumor cells (KMS26, AU565 or Saos‐2) with Matrigel (40187ES08, Yeasen, Shanghai, China) (1:1, v/v) to establish xenograft models. Tumor growth was monitored until the volumes reached 200 mm^3^. Xenograft Nude mice were randomly assigned to two groups. Mice received 50 µg of Cy5‐C33×CD16 (1 nmol) via tail vein injection, while the other group of mice 150 µg of Cy5‐BB7.2 (1 nmol). Fluorescence living imaging was performed at predetermined time points (6, 12, 24, 48, 72, 96 and 120 h) using an in vivo imaging system (IVIS) (PerkinElmer, MA, USA). The acquired images were analyzed using Living Image 4.4 software (PerkinElmer) to quantify fluorescence intensity (expressed as photons/s/cm^2^/sr) within defined tumor regions. To further analyze the in vivo targeting of Cy5‐C33×CD16, we conducted an identical parallel experiment. The mice were euthanized at 6 h post‐injection. The xenografts were resected and dissociated into single‐cell suspensions using digestion enzyme mix [87]. The single‐cell suspensions were analyzed using FCM to measure the Cy5‐positive cell population.

### 4.9 In Vivo Therapeutic Efficiency Assessment of C33×CD16 scDb

To assess the in vivo antitumor efficacy of C33×CD16 scDb, 6‐week‐old NSG mice were subcutaneously (SC) injected with 2 × 10^6^ KLE cells in the flank region. On Day 9, mice were randomized into three groups. The mice were intraperitoneally injected with isotype (1 mg/kg/dose, 20 nmol/kg/dose), hFc‐C33 (3 mg/kg/dose, 20 nmol/kg/dose) or C33×CD16 scDb (1 mg/kg/dose, 20 nmol/kg/dose) every 3 days from Day 10, each treatment group was also injected with one dose of NK92mi‐CD16a (1 × 10^7^ cells) intravenously on Day 10 and an additional dose on Day 25. No irradiation was performed on NK92mi‐CD16a cells before intravenous injection. Tumor volumes were calculated as (length × width^2^)/2 with an endpoint volume of 1500 mm^3^. At the end of experiment, xenograft tissues were dissociated into single‐cell suspensions using a digestion cocktail containing 1.5 mg/mL collagenase IV (C5138, Sigma‐Aldrich, Shanghai, China), 1.5 mg/mL collagenase I (SCR103, Sigma‐Aldrich, Shanghai, China), 1.5 mg/mL hyaluronidase (HX0514, Sigma‐Aldrich, Shanghai, China) and 0.2 mg/mL DNase I (260913, Sigma‐Aldrich, Shanghai, China). The cell suspensions were stained with APC‐labeled anti‐CD56 (17‐0567‐42, Invitrogen) at 4°C for 45 min to identify NK cells. Subsequently, the cells were stained with FITC‐labeled anti‐IFN‐γ (11‐7319‐82, Invitrogen) according to the standard intracellular staining protocol (00‐5523‐00, Invitrogen). After washing with FACS buffer, samples were analyzed using FCM (CytoFLEX LX, Beckman Coulter). NK cell infiltration (APC events) and activation (FITC‐positive in APC‐positive events) were quantified using FlowJo 10.4 software (BD Biosciences).

To further assess the in vivo antitumor efficacy of C33×CD16 scDb in combination with adoptive NK cell therapy, 6‐week‐old NSG mice were intravenously (IV) injected with luciferase‐expressing KMS26 (KMS26‐luc) (5 × 10^5^ cells) and NK92mi‐CD16a (1 × 10^7^ cells) on Day 0. No irradiation was performed on NK92mi‐CD16a cells before intravenous injection. On Day 6, mice were randomly assigned to three treatment groups: isotype (1 mg/kg/dose, 20 nmol/kg/dose), hFc‐C33 (3 mg/kg/dose, 20 nmol/kg/dose) and C33×CD16 scDb (1 mg/kg/dose, 20 nmol/kg/dose) via intraperitoneal (IP) injection every 3 days. On Day 10, all mice were injected with an additional 1 × 10^7^ NK92mi‐CD16a cells. Tumor progression was monitored using IVIS on Day 5, 10, 20. The acquired images were analyzed using Living Image 4.4 software (PerkinElmer) to quantify fluorescence intensity.

### 4.10 Data Analysis

All statistical analyses involving multiple comparisons were conducted using GraphPad Prism 10. Data are presented as mean ± standard deviation (s.d.), all data points represent biological replicates. The normality assumption for all experimental data was evaluated with the Shapiro–Wilk test. The equality of variances was verified by Brown–Forsythe test. One‐way analysis of variance (ANOVA) followed by Tukey's multiple comparisons test were utilized for comparisons among more than two groups. One‐way ANOVA followed by Dunnett's multiple comparisons test was used for pairwise comparisons between each experimental group and the designated control group. When comparing two groups, Student's *t*‐test or Student's *t*‐test with Welch's correction were applied. Nonparametric test (Kruskal–Wallis test followed by Dunn's multiple comparisons test) was used when the data did not meet the assumption of normality. Statistical significance was calculated using GraphPad Prism 10 or Microsoft Excel (2021). Survival analysis was performed using the Kaplan–Meier method with the log‐rank test. A *p*‐value less than 0.05 was considered statistically significant. FCM data were analyzed using FlowJo version 10.4, including the generation of dot plots. Half‐life time was analyzed by fitting data to either a single‐phase or a biphasic exponential decay model using GraphPad Prism 10. Fluorescence microscopy images were analyzed using Olympus FV31S‐SW Viewer (Olympus), and relative fluorescence intensities (RFIs) were quantified with ImageJ software (LOCI, University of Wisconsin). In vivo fluorescence imaging in mice was analyzed using Living Image 4.4 (PerkinElmer) to determine average Cy5 signal intensity (expressed as p/sec/cm^2^/sr) within selected region of interest (ROIs). All tumor measurements and image quantification were performed by investigators blinded to experimental group assignments. DNA sequence alignments were performed using NCBI's Nucleotide BLAST tool. For surface plasmon resonance (SPR) experiments, binding curves were analyzed with Biacore Insight Evaluation Software (GE Healthcare, Marlborough, MA) based on a 1:1 interaction model.

## Author Contributions

Guangli Suo: Conceptualization, Methodology, Funding acquisition, Project administration, Supervision, Validation and Writing – review & editing; Hanfei Wu: Conceptualization, Data curation, Methodology, Investigation, Software, Formal analysis, Validation, Writing – original draft, Writing – review & editing; Zhe Zhao, Zhongjuan Xu: Project administration, Funding acquisition, Supervision; Xuan Xiong, Mengdie Yang, Weiping Ge, Danyue Zhi, Xiaobin Cao and Chuanqian Yi: Investigation; Yong Qiao, Junjun Cao, Yan Mei and Shiping Jiao: Technical support; Quan Liu, Li Zhang and Hao Zhou: Funding acquisition, Clinical resources. All authors have read and approved the final manuscript.

## Funding

This research was supported by funds from the Jiangsu Province's Major Science and Technology Project (grant no.: BG2025046), the Natural Science Foundation of Jiangsu Province (grant no.: BK20250510), the National Natural Science Foundation of China (grant nos.: 32470814 and 82202345), the Natural Science Foundation of Jiangsu Province (grant no.: BK20210126), the Youth Foundation Project from Guangdong Basic and Applied Basic Research Foundation (grant no.: 2022A1515110885), the National Key Research and Development Program of China (grant no.: 2022YFC2705005), and the Shanghai Science and Technology Committee (grant no.: 24SF1904700). This study was supported by LivingChip and Sinano Co‐operating Joint Laboratory for Single‐cell Technology Research. The funders had no role in study design, data collection and analysis, interpretation of the data, writing of the report, or in the decision to submit the article for publication.

## Conflicts of Interest

Yong Qiao, Junjun Cao, and Yan Mei are the employees in Nanjing LivingChip Biotechnology Co. Ltd. Shiping Jiao is an employee in TCRX Therapeutics (KeShiHua) Co. Ltd, but has no potential relevant financial or non‐financial interests to disclose. The other authors declare no conflicts of interest.

## Ethics Approval

All procedures and studies involving animals were carried out in compliance with a protocol that received approval from the Animal Care and Use Committee at the Suzhou Institute of Nano‐Tech and Nano‐Bionics, Chinese Academy of Sciences (Protocol Number: SINANO/EC/2025‐109). All human samples were used for non‐interventional study. All human samples were obtained after the donors had been fully informed and had provided their written informed consent. The study protocol was approved by the Institutional Review Board of the First Affiliated Hospital of Soochow University (ethical official number: (2023) No. 249), adhering to the principles stated in the Declaration of Helsinki.

## Supporting information




**Supporting file 1**: mco271058‐sup‐0001‐SuppMat.docx

## Data Availability

All data and materials that support the study's findings are available in the article and its supporting files, or from the corresponding author on reasonable request.
