# Supplementary material for: Bispecific NK‐Cell Engager Targeting TP53 Mutation Through Integration of a TCR‐mimic Antibody
Source: MedComm (2020). 2026 Oct 15;7(11):e71058. doi: 10.1002/mco2.71058 (PMC13669819; doi:10.1002/mco2.71058)
Supplement: Supplementary file 1 [file MCO2-7-e71058-s001.docx]

**Supplementary information**

**Bispecific NK-Cell Engager Targeting TP53 Mutation through Integration of a TCR-mimic Antibody**

Hanfei Wu^1, 2^, Zhe Zhao^2, 3^, Li Zhang^4^, Xuan Xiong^1, 2^, Mengdie Yang^1, 2^, Weiping Ge^1, 2^, Danyue Zhi^1, 2^, Xiaobin Cao^1, 2^, Chuanqian Yi^1, 2^, Yong Qiao^5^, Junjun Cao^5^, Yan Mei^5^, Shiping Jiao^6, 7^, Quan Liu^8, 9^, Hao Zhou^10^, Zhongjuan Xu^2*^, Guangli Suo^1, 2, 3*^

**^1^** School of Nano-Tech and Nano-Bionics, University of Science and Technology of Chin(A) Hefei, Anhui, 230026, China.

**^2^** CAS Key Laboratory of Nano-Bio Interfac(A) Suzhou Institute of Nano-Tech and Nano-Bionics, Chinese Academy of Sciences, Jiangsu, 215123, China.

**^3^** Department of Pharmaceutical Engineering, School of Engineering, China Pharmaceutical University, Nanjing, Jiangsu, China.

**^4^** Department of Vaccine Clinical Evaluation, Jiangsu Provincial Center for Disease Prevention and Control, Nanjing 210009, China.

**^5^** Nanjing LivingChip Biotechnology Co., Lt(A) Nanjing, 211800, China.

**^6^** Clinical Research War(A) The Sixth People’s Hospital, Shanghai Jiao Tong University, Shanghai, 200233, China.

**^7^** TCRX Therapeutics (KeShiHua) Co. Lt(A) Nanjing, 210018, China.

**^8^** Department of Otolaryngology, Ey(A) Ear, Nose and Throat Hospital, Fudan University, Shanghai, 200031, China.

**^9^** Skull Base Adenoid Cystic Carcinoma Center, Ey(A) Ear, Nose and Throat Hospital, Fudan University, Shanghai, 200031, China.

**^10^** Department of General Surgery, The First Affiliated Hospital of Soochow University, Suzhou, 215006, China.

* Corresponding authors:

E-mail addresses: glsuo2013@sinano.ac.cn (Guangli Suo); zjxu2015@sinano.ac.cn (Zhongjuan Xu)

1. **Supplementary Figures**

**Figure S1. Characterization of the HMSC-chip and single-cell array.** (A) Image of the HMSC-chip. (B) Image of the single-cell array on HMSC-chip.

**Figure S2. Confirmation of p53^R175H^/HLA-A*02:01-specific targeting of potential TCRm-Abs.** (A) Cross-reactivity of antigen peptides for five selected clones, analyzed by T2 binding assay detected *via* FCM. Six different control peptides were used to generate control T2 cells. Representative results (A) and statistical results (B) demonstrate the cross-reactivity of five potential TCRm-Abs. (C) SPR analysis results of C33-Ab binding to p53^WT175^/HLA-A*02:01 or p53^R175H^/HLA-A*02:01. SPR analysis results of C71-Ab (D), C115-Ab (E), C142-Ab (F), C171-Ab (G) binding to p53^WT175^/HLA-A*02:01 or p53^R175H^/HLA-A*02:01. *K*_D_: dissociation constant. (H) Representative histograms from FCM analysis of alanine-scanning assay. T2 cells loaded alanine-scanning peptides were stained with C33-Ab. (I) Statistical analysis of relative fluorescence intensity (RFI) values for C33-Ab binding to T2 cells loaded alanine-scanning peptides (presented by T2 cells). (J) Statistical analysis of RFI values for C33-Ab binding to ZBTB10 and IAP peptides (presented by T2 cells). Data represent mean ± s.d.; n = 3 biological replicates.

**Figure S3. Bispecific binding of scDbs and their binding stability.** Bispecific binding of scDbs was confirmed using FCM using T2-p53^R175H^ cells (A) and NK92mi-CD16a cells (B) as targets. Surface retention was assessed at different time points after binding to T2-p53^R175H^ cells (C) and NK92mi-CD16a cells (D). Binding ability of scDbs was evaluated after their incubation in serum for varying durations using T2-p53^R175H^ cells (E) and NK92mi-CD16a cells as targets (F). Data are presented as means ± s.d. (n =3 biological replicates). (G) Western blotting showed that purified C33×CD16 has a molecular weight of ~53 kDa. (H) The purified scDb was analyzed using size exclusion chromatography (SEC). UV absorbance chromatograms at 280 nm revealed a major peak eluting at 13.2 minutes. (I) Differential scanning fluorimetry showed a melting temperature (*T*_m_) of 62.2°C for C33×CD16. SPR analysis of C33×CD16 binding to p53^R175H^/HLA-A*02:01 (J) and to p53^WT175^/HLA-A*02:01 (K). *K*_D_: dissociation constant.

**Figure S4. *In vivo* targeting of Cy5-labeled C33×CD16.** (A) Nude mice xenograft models (KMS26, AU565, Saos-2) were established and intravenously injected with Cy5 labeling C33×CD16 (Cy5-C33×CD16). Images were captured at 0 hour, 6 hours, 24 hours, 48 hours, 72 hours, 96 hours and 120 hours timepoints using IVIS to monitor signal distribution. Dashed circles indicate xenograft locations. (B) Quantitative analysis of the signal intensities is presented as curve plots. Analysis of xenograft-derived single-cell suspensions using FCM to measure Cy5 fluorescence-stained cells. Representative result (C) and statistic result (D). Data represent mean ± s.d.; n = 3 biologically independent mice.

**Figure S5. NK cell-mediated cytotoxicity induced by C33×CD16.** (A) Gating strategy for flow cytometry analysis. Representative dot plots show that the PI-positive in CFSE-positive target cell population were regarded as dead cells resulting from NK cell-mediated cytotoxicity. (B) ELISA analysis of supernatants shows secretion levels of IFN-γ, TNF-α, Granzyme-B, Perforin and IL-2. Statistical significance was determined by unpaired Student’s *t* test with Welch’s correction. “*”: P < 0.05, “**”: P < 0.01, “***”: P < 0.001. (C) Confocal fluorescent images of different tumor cells co-cultured with NK92mi-CD16a cells at an E:T ratio of 10:1 to evaluate NK cell-mediated cytotoxicity. Cells were treated with isotype scDb hFc-C33 or C33×CD16 (5×10^-8^ M). Representative images were captured 24 hours post-treatment. Red: PI staining; Green: Calcein AM staining. (D) Statistical results showing the cytotoxicity elicited by primary NK cells toward primary tumor cells in the presence of C33×CD16. NK-mediated cytotoxicity was assessed based on dead cell counts (PI-positive cells). (E) Representative dot plots show the percentage of PI-positive KMS26 cells in co-culture assay using primary NK cells as effector cell. (F) Statistic result showing the cytotoxicity elicited by primary NK cells toward KMS26 in the presence of C33×CD16 or hFc-C33 (5×10^-8^ M). (G) Statistic result showing the cytotoxicity elicited by primary NK cells toward different cell lines in the presence of C33×CD16 or hFc-C33 (5×10^-8^ M). Data represent mean ± s.d.; n = 3 biological replicates. Statistical significance was determined by one-way ANOVA followed by Tukey’s multiple comparisons test. “*”: P < 0.05, “**”: P < 0.01, “***”: P < 0.001, ns: not significant, P > 0.05.

**Figure S6. Generation of micro-tumorspheres (MTSs) derived from primary tumor tissues.** (A) Schematic illustration of the MTS generation workflow. Created in BioRender. Wu, H. (2026) <https://BioRender.com/3povypo.> (B) Representative image of the HMWC-chip featuring microwells with an 80-µm diameter. Representative bright-field micrographs of MTSs formation over time on the HMWC-chip: Patient #1 (C), Patient #2 (D), Patient #3 (E) and Patient #4 (F) captured at 1, 3, and 7 days post cell loading. (G) Representative histograms of FCM analysis that confirming the expression of HLA-A*02:01 and the presentation of p53^R175H^/HLA-A*02:01 in patient samples. BB7.2: anti-HLA-A2 antibody, H2: anti-p53^R175H^/HLA-A*02:01 antibody.

**Figure S7. Risk assessment of CRS using PBMCs co-culture assay.** Statistic results showing the secreting level of CRS marker cytokine by PBMCs in the presence of different reagents: Fab Ctrl., C33×CD16, LPS or TGN1412. Data represent mean ± s.d.; n = 3 biological replicates. Statistical significance was determined by one-way ANOVA followed by Dunnett’s multiple comparisons test (all groups vs. C33×CD16). “*”: P < 0.05, “***”: P < 0.001, ns: not significant, P > 0.05.

2. **Supplementary Tables**

**Table S1.** The sequences of peptides used in T2 binding assay or alanine scanning assay.

| **Peptide** | **Sequence** |
| --- | --- |
| p53^R175H^ | HMTEVVRHC |
| p53^WT^ | HMTEVVRRC |
| p53^A9^ | HMTEVVRHA |
| p53^A8^ | HMTEVVRAC |
| p53^A7^ | HMTEVVAHC |
| p53^A6^ | HMTEVARHC |
| p53^A5^ | HMTEAVRHC |
| p53^A4^ | HMTAVVRHC |
| p53^A3^ | HMAEVVRHC |
| p53^A2^ | HATEVVRHC |
| p53^A1^ | AMTEVVRHC |
| Survivin | RISTFKNWPFL |
| Flu | GILGFVFTL |
| WT-1 | RMFPNAPYL |

**Table S2.** A bioinformatic scan of the human proteome for HLA-A*02:01-presented peptides homologous to HMTEVVRHC.

| **Top 3 Expressed Tissues^4^** | Thymus; prostate; trachea | Brain, cerebellum; lung; brain, Whole | Placenta; thymus; adrenal gland | Thyroid; kidney; spleen | Prostate; brain, cerebellum; placenta | Thyroid; prostate; thmus |
| --- | --- | --- | --- | --- | --- | --- |
| **Score^3^** | 22.3 bits (45) | 21.4 bits (43) | 19.7 bits (39) | 19.7 bits (39) | 18.0 bits (35) | 17.2 bits (33) |
| **Identitiy^2^** | 6/9 (67%) | 7/9 (78%) | 6/9 (67%) | 6/9 (67%) | 5/9 (56%) | 5/9 (56%) |
| **%Rank_EL (HLA-A*02:01)^1^** | 7.525 | 70.000 | 16.280 | 8.000 | 0.605 | 0.659 |
| **Sequence** | HVTEVVKRC | MHEVVRKHC | HMKYVVRRC | QMTE-GRHC | QMSEVVQTC | QLTEVVHLC |
| **UniProt ID** | Q9H3D4 | Q96G74 | Q9BYJ4 | Q9Y2L6 | Q96DT7 | Q9Y573 |
| **Gene ID** | 8626 | 55593 | 53840 | 23150 | 65986 | 3652 |
| **Source protein** | Tumor protein 63 | OTU domain-containing protein 5 | Tripartite motif containing 34 | FERM domain-containing protein 4B | Zinc finger and BTB domain-containing protein 10 | Intracisternal A particle-promoted  polypeptide |

^1^ %Rank_EL is the HLA-A*02:01 ligand rank predicted by the NetMHCpan 4.1 EL model; the smaller the value, the higher the binding potential. %Rank_EL (HLA-A*02:01) of HMTEVVRHC (p53^R175H^) is 1.761;

^2^ Identity refers to the number of fully matched amino acid residues with the target epitope HMTEVVRHC (p53^R175H^)；

^3^ Score is the bitscore of BLASTp alignment;

^4^ Gene expression levels were from a public total RNA-seq dataset covering 20 human tissues (BioProject: PRJNA280600).

**Table S3.** The Aa sequences of the variable region of 3G8 used in this study.

| **Antibody** | **Aa sequences** |
| --- | --- |
| 3G8-H | QVTLKESGPGILQPSQTLSLTCSFSGFSLRTSGMGVGWIRQPSGKGLEWLAHIWWDDDKRYNPALKSRLTISKDTSSNQVFLKIASVDTADTATYYCAQINPAWFAYWGQGTLVTVSA |
| 3G8-L | IVLTQSPASLAVSLGQRATISCKASQSVDFDGDSFMNWYQQKPGQPPKLLIYTTSNLESGIPARFSASGSGTDFTLNIHPVEEEDTATYYCQQSNEDPYTFGGGTKLELKR |

**Table S4.** The genotyping of HLA-A using seuqencing-based typing (SBT).

| **Cell line** | **HLA-A subtype** |
| --- | --- |
| KMS26 | 02:01 |
|  | 31:01 |
| KLE | 02:01 |
|  | 24:02 |
| AU565 | 31:01 |
|  | 03:01 |
| Saos-2 | 02:01 |
|  | 24:02 |
| AU565-HLA | 02:01 |
|  | 03:01 |
| Saos-2-p53^R175H^ | 02:01 |
|  | 24:02 |
| KLE^tp53KO^ | 02:01 |
|  | 24:02 |

**Table S5.** The p53^R175H^/HLA-A*02:01 expression of cell lines used for specificity test.

| **Cell line** | **HLA-A*02:01** | **p53^R175H^** |
| --- | --- | --- |
| KMS26 | ＋ | ＋ |
| KLE | ＋ | ＋ |
| AU565 | - | ＋ |
| Saos-2 | ＋ | - |
| AU565-HLA | ＋ | ＋ |
| Saos-2-p53^R175H^ | ＋ | ＋ |
| KLE^tp53KO^ | ＋ | - |

**Table S6.** The clinical informations of patient samples.

| **Patient ID** | Patient 1 | Patient 2 | Patient 3 | Patient 4 |
| --- | --- | --- | --- | --- |
| **Gender** | Female | Female | Female | Female |
| **Age (years)** | 58 | 44 | 41 | 53 |
| **Histology** | Invasive ductal carcinoma | Invasive ductal carcinoma | High grade intraductal carcinoma with lobular carcinoma | Invasive ductal carcinoma |
| **Molecular pathology** | HER2+ | Triple negative | HER2+ | HER2+ |
| **Grading** | III | III | NA | NA |
| **Distant metastasis** | No | No | No | No |
| **Neoadjuvant chemotherapy (NAC)** | Yes | Yes | No | Yes |
| **Recurrence** | No | Yes | No | No |
| **ABO blood type** | B+ | A+ | O+ | O+ |
| **HLA-1A** | 02:01 | 02:01 | 02:01 | 02:01 |
| **HLA-2A** | 02:07 | 11:01 | 11:01 | 30:01 |
| **HLA-1B** | 13:02 | 38:02 | 15:11 | 38:02 |
| **HLA-2B** | 35:03 | 51:01 | 54:01 | 46:01 |
| **HLA-1C** | 04:01 | 07:02 | 01:02 | 01:02 |
| **HLA-2C** | 06:02 | 14:02 | 03:03 | 07:02 |
| **P53^R175H^** | + | - | + | - |

**Table S7.** The sequences of primers used in this study.

| Primers | Sequences | Applications |
| --- | --- | --- |
| HLA-F | gattctagagctagcATGGCCGTCATGGCGCCC | HLA-A2 clone |
| HLA-R | atccttcgcggccgcTCACACTTTACAAGCTGTGAGAGACA |  |
| p53-F | gattctagagctagcATGGAGGAGCCGCAGTCAG | p53 clone |
| p53-R | atccttcgcggccgcTCAGTCTGAGTCAGGCCCTTCT |  |

3. **Supplementary Methods**

**3.1 Cell lines**

Saos-2 (ATCC: CRL-7939) cell line and AU565 (ATCC: CRL-2351) were provided by Meisen cell technology Co. Ltd. (Hangzhou, China). The KMS26 (JCRB1187) was provide by HonSun Biological Technology Co., Ltd (Shanghai, China). The NK92mi (ATCC: CRL-2408), KLE (ATCC: CRL-1622) and SP2/0 (ATCC: CRL-1581) were provide by Procell (Wuhan, China). *TP53* gene was disrupted to obtain KLE^tp53KO^ cell line using CRISPR/Cas9 conducted by GENEWIZ (Suzhou, China). The T2 (ATCC: CRL-1992) was obtained from Genetimes ExCell Technology (Shanghai, China). The T2, AU565, hybridomas and SP2/0 cell lines were grown in RPMI-1640 medium containing 10% fetal bovine serum (FBS) and 1% penicillin-streptomycin (P/S). For hybridomas culture, the RPMI-1640 medium was further supplemented with 2% hybridoma fusion and cloning supplement (HFCS). The KMS26, HEK293T, and Saos-2 cell lines were cultured in Dulbecco's modified eagle medium (DMEM) containing 10% FBS and 1% P/S. The KLE and KLE^tp53KO^ cells were cultured in DMEM/F-12 supplemented with 15% FBS and 1% P/S. The NK92mi-CD16a (158V) cell line was cultured in α-MEM complete medium containing 12.5% horse serum (Procell, Wuhan, China) and 1% P/S, further supplemented with inositol (0.2 mM), β-mercaptoethanol (0.1 mM), and folic acid (0.02 mM) to ensure optimal growth conditions. The HLA genotype and p53 mutation status of the KMS26, AU565, Saos-2, and KLE cell lines are described in Supplementary Table S4 and S5. All culture medium, including RPMI-1640, DMEM, and DMEM/F-12, were obtained from GIBCO (ThermoFisher, Waltham, MA, USA). FBS and P/S supplements were also sourced from GIBCO (ThermoFisher, Waltham, MA, USA). The HFCS for hybridomas culture was purchased from Roche (Basel, Switzerland). The α-MEM, inositol, β-mercaptoethanol, and folic acid were purchased from Pricella (Wuhan, China). All cell lines were maintained in humidified incubator at 37°C with 5% CO_2_.

**3.2 Mouse immunization and hybridoma generation**

For mouse immunization, six-week-old BALB/c mice were intraperitoneally (IP) injected with the pHLA complex p53^R175H^/HLA-A*02:01 as the antigen, which emulsified with Freund’s complete adjuvant (D614-0050, Beyotime, Shanghai, China) for the primary immunization. Three booster injections were administered every 10 days. Blood samples were collected from the orbital sinus 3 days after each immunization and used for antibody titer detection by Enzyme-linked immunosorbent assay (ELISA) to confirm a successful immune response. A final booster dose was administered 3 days before the fusion of SP2/0 cells with splenocytes. On the day of cell fusion, the immunized mice were euthanized, and splenocytes were harvested. These splenocytes were fused with SP2/0 myeloma cells at a 1:1 number ratio using polyethylene glycol (PEG) (P7181, Sigma-Aldrich, Burlington, MA, USA). The fused cells were then cultured in RPMI-1640 medium supplemented with HFCS (Roche, Basel, Switzerland) and hypoxanthine-aminopterin-thymidine (HAT) (H0262, Sigma-Aldrich, Burlington, MA, USA) to selectively support hybridomas growth. After 3 days of culture, the medium was refreshed. By day seven, the resulting hybridomas (p53^R175H^/HLA-hybridomas) were collected for screening TCRm-ABs specifically targeting p53^R175H^/HLA-A*02:01.

**3.3 Enzyme-linked immunosorbent assay (ELISA)**

The ELISA was conducted to measure antibody titers in serum from immunized mice or purified single-chain diabody (scDb) targeting the p53^R175H^/HLA-A*02:01 antigen. A 96-well plate was coated with 100 μL per well of p53^R175H^/HLA-A*02:01 (1 μg/mL) and incubated overnight at 4°C. The coating solution was then removed, and the wells were blocked with blocking buffer (PBS containing 0.01% Tween-20, 3% BSA) for 1 hour at 37°C, followed by three washes with PBST (PBS with 0.01% Tween-20). For detection of scDb or isotype specificity, the plates were incubated with serial dilutions of scDbs or antibodies at room temperature for 1 hour. After three washes with PBST, scDb detection was carried out by incubating with 1 µg/mL recombinant protein L (77679, ThermoFisher Scientific) for 1 hour at room temperature, followed by incubation with an anti-protein L horseradish peroxidase (HRP) conjugate (ab63506, Abcam, Shanghai, China) at a 1:10, 000 dilution for an additional hour. For serum antibody detection, diluted samples were added to the coated plate (100 μL/well), and Goat Anti-Mouse IgG H&L (HRP) (ab205719, Abcam, Shanghai, China) was used as the secondary antibody. Following three washes with PBST, 50 μL of 3,3’,5,5’-tetramethylbenzidine (TMB ELISA substrate) (ab171523, Abcam, Shanghai, China) was added to initiate the colorimetric reaction. The reaction was stopped after 10 minutes by adding 50 μL of 1 M H_2_SO_4_, and the absorbance was read at 450 nm using a microplate reader (Cytation 3, BioTek).

For the detection of cytokines in cell culture supernatants, five commercial ELISA kits were used to quantify cytotoxic factors secreted by NK92mi-CD16a (158V) cells, including IFN-γ (PI511, Beyotime, Shanghai, China), IL-2 (E-EL-H0099, Elabscience, Wuhan, China), perforin (BMS2306, Thermofisher Scientific, MA, USA), granzyme B (GzmB, E-EL-H1617, Elabscience, Wuhan, China), and TNF-α (88-7346-88, Thermofisher Scientific, MA, USA), following the manufacturers’ instructions.

For the detection of cytokines in peripheral blood mononuclear cells (PBMCs) culture supernatants to evaluate the CRS potential, four commercial ELISA kits were used to quantify cytokines secreted, including IFN-γ (PI511, Beyotime, Shanghai, China), IL-6 (E-EL-H6156, Elabscience, Wuhan, China), IL-8 (E-EL-H6008, Elabscience, Wuhan, China), and TNF-α (RGH810-2, Real-gen Biotechnology, Jiangsu, China), following the manufacturers’ instructions.

**3.4 Antibody gene sequencing**

Monoclonal hybridoma cells producing target-specific antibodies were initially expanded in 96-well plates and subsequently transferred to 6 cm culture dishes. Upon reaching near-confluence (approximately 90%), the cells were gently dissociated, collected by centrifugation at 200 g for 5 minutes, and resuspended in RNA extraction buffer. Total RNA was isolated using a commercial extraction reagent Total RNA Extraction Reagent (R401-01, Vazyme, Nanjing, China), followed by first-strand cDNA synthesis using a reverse transcription kit (HiScript 1st Strand cDNA Synthesis Kit, R111-01, Vazyme, Nanjing, China). The variable regions of immunoglobulin genes were amplified by PCR using degenerate primers targeting murine IgG heavy and light chain sequences. The amplified products were purified using PCR Purification Kit (19106ES50, Yeasen, Shanghai, China) and sequenced by GENEWIZ (Suzhou, China). Sequence analysis was performed using the National Center for Biotechnology Information (NCBI) IGBLAST tool for immunoglobulin gene characterization (https://www.ncbi.nlm.nih.gov/igblast/).

**3.5 Construction of expression and viral vectors**

C33-Ab is a mouse IgG2b antibody derived from C33 hybridomas. hFc-C33 is a recombinant antibody containing the identical Fab fragment of C33-Ab but substituting the murine Fc fragment with a human IgG1 Fc domain, targeting the p53^R175H^/HLA-A*02:01 complex. The Fab fragment of C33-Ab served as the targeting module for NKCE construction. To produce the recombinant antibody (hFc-C33) containing the variable region of C33-Ab and the human IgG1 constant region, expression vectors were constructed according to the instructions provided by the ClonExpress MultiS One Step Cloning Kit (C113-01, Vazyme, Nanjing, China). Briefly, synthetic variable region sequences were ligated into mammalian expression vectors carrying human IgG constant domains. The heavy chain variable region (VH) was cloned into the pcDNA3.4-H vector (containing the human IgG1 constant region), while the light chain variable region (VL) was inserted into the pcDNA3.4-k vector (containing the human κ-light chain constant region).

The C33×CD16 recombinant gene was designed and constructed by GENEWIZ (Suzhou, China) using the following elements from N- to C-terminus: an IL-2 signal peptide, the VL of anti-p53^R175H^/HLA-A*02:01 (C33), a GGGGS linker, the VH of anti-CD16a (3G8), a (GGGGS)_3_ linker, the VL of anti-CD16a (3G8), another GGGGS linker, the VH of anti-p53^R175H^/HLA-A*02:01 (C33), and a 6×His tag. The rC33×CD16 gene, a reversed version of C33×CD16 scDb, had the following order: IL-2 signal peptide, the VL of anti-CD16a (3G8), a GGGGS linker, the VH of anti-p53^R175H^/HLA-A*02:01 (C33), a (GGGGS)_3_ linker, the VL of anti-p53^R175H/^HLA-A*02:01 (C33), another GGGGS linker, the VH of anti-CD16a (3G8), and a 6×His tag. In the isotype control scDb, the variable regions of a irrelevant antibody from an other project aiming at obtain WT-1/HLA-A*02:01 TCRm-Ab (confirmed non-reactive against p53^R175H^/HLA-A*02:01 and HLA-A*02:01) were constructed with the variable regions of anti-CD16a (3G8) as described above. The genes C33×CD16 and rC33×CD16 were cloned into the pcDNA3.4 vector (pcDNA3.4-C33×CD16 and pcDNA3.4-rC33×CD16, respectively) and all constructs were validated by sequencing prior to expression analysis.

The HLA-A*02:01 and p53^R175H^ genes were individually inserted into the pCDH-CMV-MCS-EF1-GFP+Puro backbone (Addgene) *via* *Eco*RI and *Bam*HI restriction sites to generate the lentiviral constructs pCDH-CMV-HLA-A*02:01 and pCDH-CMV-p53^R175H^. The primers used for cloning were designed to include homology arms according to the ClonExpress MultiS One Step Cloning Kit protocol (C113-01, Vazyme, Nanjing, China), with sequences detailed in Supplementary Table S7. Following transformation into DH5α competent cells for plasmid amplification, all constructs were validated by Sanger sequencing.

**3.6 Production and purification of recombinant antibodies**

For recombinant antibody production, HEK293F cells were co-transfected with expression vectors pcDNA3.4-H and pcDNA3.4-k using a commercial transfection system (A14524, ThermoFisher Scientific, MA, USA). Cells were seeded at 3×10^6^ cells/mL in 25 mL culture volume in 125 mL culture flask. Plasmid DNA (25 μg heavy chain and 25 μg light chain constructs) was separately complexed with lipofectamine transfection reagent in Opti-MEM medium following the manufacturer’s protocol. The lipid-DNA complexes were added to the cell culture in a CO_2_ incubator with orbital shaking (100 rpm). Transfection enhancers (A14524, GIBCO, ThermoFisher, Waltham, MA, USA) were added after 24 hours to boost antibody expression. After 6 days of culture, the culture supernatants were harvested, centrifuged, and filtered (0.2 μm). Antibodies were purified by protein G affinity chromatography (P2015, Beyotime, Shanghai, China) using a 30 mL column equilibrated with PBS (pH 7.4) containing 150 mM NaCl. Filtered supernatants were loaded onto the column for Fc domain-mediated antibody capture. Following PBS wash, bound antibodies were eluted with 0.1 M glycine buffer (pH 2.75) and immediately neutralized with 1 M Tris-HCl (pH 8.8). Purified antibody concentration was quantified by ELISA, and the final product was stabilized in PBS containing 50% glycerol for storage at -20°C.

**3.7 scDb expression and purification**

The scDbs was expressed using a commercial transfection system (A14524, ThermoFisher Scientific, MA, USA). HEK293F cells were seeded at a density of 3×10^6^ cells/mL in a total culture volume of 25 mL in 125 mL culture flask. The pcDNA3.4-C33×CD16 or pcDNA3.4-rC33×CD16 plasmid was diluted in 1.5 mL Opti-MEM medium to make a final concentration of 1 μg/mL, and 500 μL lipofectamine was similarly diluted in another 1.5 mL Opti-MEM medium. These two solutions were then mixed and incubated for 15 minutes at room temperature to allow the formation of lipid-DNA complexes. Subsequently, the lipid-DNA complexes were added to the cell culture with gentle mixing *via* orbital shaking (100 rpm) in a CO_2_ incubator. Transfection enhancers (A14524, GIBCO, ThermoFisher, Waltham, MA, USA) were added 24 hours post-incubation at 37°C to boost antibody expression. Following a 6-day incubation, the culture supernatant was harvested and filtered through 0.22 μm Syringe Filters (FF342-10pcs, Sartorius, Shanghai, China).

Purification was performed using NTA-Ni Magnetic Agarose Beads (P2241-2ml, Beyotime, Shanghai, China) for His-Tag protein purification. Briefly, the NTA-Ni beads were gently washed 3 times with washing buffer (10 mM Tris, 500 mM NaCl, 20 mM Imidazole, pH 7.4) and collected using a magnetic rack. Subsequently, the NTA-Ni beads were incubated with filtered culture supernatant for 35 minutes at room temperature on a rotary mixer. Following incubation, the mixture was placed on a magnetic rack for 20 seconds to collect the beads, and the remaining liquid was carefully removed. The NTA-Ni beads were then subjected to 3 additional washes with 1 mL of washing buffer. Finally, 1 mL of elution buffer (10 mM Tris, 500 mM NaCl, 500 mM Imidazole, pH 7.4) was added to the NTA-Ni beads, followed by incubation for 5 minutes with gentle inversion every 30 seconds. The purified scDbs (C33×CD16 or rC33×CD16) was subsequently recovered by placing the mixture on a magnetic rack for 20 seconds, allowing the eluate to be collected.

**3.8 Western blot**

Protein purity was quantified using UV Spectrophotometry based on the A280/A260 ratio. The concentration of purified scDb or antibody was quantified using a BCA protein assay kit (23227, ThermoFisher Scientific, MA, USA). Equal amounts of protein (20 µg per lane) were separated by electrophoresis on 12% precast gels (P0050A, Beyotime, Shanghai, China) and subsequently transferred onto PVDF membranes (FFP24, Beyotime, Shanghai, China). Following three washes with PBST, the membrane was incubated with Western Blocking Solution (P0023B-100ml, Beyotime, Shanghai, China) for 15 minutes at room temperature. After removing the blocking solution and an additional wash with PBST, the membranes were incubated with HRP-conjugated anti-6×His tag primary antibody (ab1187, Abcam, Shanghai, China) at dilution of 1:3000 in Western Primary Antibody Dilution Solution (P0023A-100ml, Beyotime, Shanghai, China) overnight at 4°C. After three washes with PBST, the membrane was treated with ECL Chemiluminescent Substrate (36222ES60, Yeasen, Shanghai, China) for 5 minutes at room temperature, and the protein bands were visualized using a gel imaging system (Amersham Imager 600, GE Healthcare).

**3.9 Surface plasmon resonance analysis (SPR)**

The binding kinetics of purified antibodies (TCRm antibodies or scDbs) to p53^R175H^/HLA-A*02:01 complexes were determined using SPR with Biacore T200 (Cytiva, Shanghai, China). A CM5 sensor chip (29149603, Cytiva, Shanghai, China) was conditioned using 10 mM sodium acetate buffer (pH 5.0) as the conjugating buffer. The chip surface was activated by injecting a freshly prepared solution containing 11.5 mg/mL N-hydroxysuccinimide (NHS) (56480, Sigma-Aldrich, Shanghai, China) and 75 mg/mL 1-ethyl-3-(3-dimethylaminopropyl) carbodiimide (EDC) (E7750, Sigma-Aldrich, Shanghai, China) for 7 minutes. TCRm antibodies (5 μg/mL in 10 mM acetate buffer, pH 5.0) were then injected into the flow cell. Remaining reactive groups were subsequently blocked by injecting 1 M ethanolamine (pH 8.5) for 7 minutes. Serial dilutions of p53^R175H^/HLA-A*02:01 pHLA (ranging from 1.56 to 50 μg/mL in running buffer) were injected for 180 seconds of association time, followed by a dissociation phase monitored for 300 seconds. Data were processed and analyzed using Biacore T200 evaluation software to determine the binding affinity (*K*_D_), association rate constants (ka) and dissociation rate constants (kd).

**3.10 Differential scanning fluorimetry (DSF)**

The thermal stability of the scDb was assessed using a DSF assay, which monitors the fluorescence of a dye that binds to exposed hydrophobic regions of the protein during heat-induced unfolding [71]. The assay was performed in a 20 μL reaction volume prepared in a whites low-profile 96-well PCR plate (MQ51501S, Monad, SF, USA). The reaction mixture consisted of 2 μL of purified scDb (1 mg/mL, final concentration 0.1 mg/mL) and 2 μL of 50×AGSYPRO Orange (AG31002, Accurate Biology, Changsha, China) in PBS (pH 7.2). After sealing the plate with an optically clear film (MP71001, Monad, SF, USA), the plate was centrifuged at 300 g for 30 seconds and then analyzed using a real-time PCR system (LightCycler 480 II, Roche). The melting temperature (*T*_m_) was determined using LightCycler 480 software.

**3.11 Binding stability of scDbs**

The cell surface retention of binding scDbs was assessed according to the previously reported protocols^44^. In brief, NK92mi-CD16a and KMS26 (3×10^5^ cells each) were incubated separately in a 96-well plate for 1 hour on ice with C33×CD16 or rC33×CD16 at a concentration of 2 µg/mL. Following incubation, the cells were washed three times with 200 µL of cold FACS buffer (PBS, 1% FBS, 0.1% sodium azide, pH 7.2) and centrifuged. The cells were then resuspended in 200 µL of FACS buffer and transferred to a 37°C incubator. At designated time intervals, the cells were washed three times with 200 µL of FACS buffer and collected for staining with 6×His Tag Monoclonal Antibody-AF488 (MA1-21315-A488, ThermoFisher Scientific, MA, USA). Surface-retained molecules were quantified using FCM (CytoFLEX LX, Beckman Coulter). The measurements were normalized to data from the initial time point (*t*_0_=100%). The half-life of cell surface retention was determined by fitting the data to either a single-phase or a biphasic exponential decay model.

To evaluate scDbs stability in serum, C33×CD16 or rC33×CD16 was incubated at a concentration of 2 µg/mL in 200 µL of human serum derived from a healthy doner at 37°C. At different time points, residual binding activity was measured using FCM. Residual binding activity at the initial time point was defined as 100%, and subsequent values were normalized to it. The half-life of each binding site was determined by fitting the data to a one-phase exponential decay model.

**3.12 Lentivirus production and stable cell lines construction**

For lentivirus production, HEK293T cells were co-transfected with either pCDH-CMV-HLA-A*02:01 to produce HLA-A*02:01-expressing virus or pCDH-CMV-p53^R175H^ to produce p53^R175H^-expressing virus, along with the packaging plasmids pVSVG (Addgene: 138479), pCMVR8.74 (Addgene: 22036), and pRSV-Rev (Addgene: 12253), using Lipofectamine (18324012, ThermoFisher Scientific, MA, USA). Viral supernatants were collected 48 hours post-transfection and used to transduce target cells. Saos-2 cells were transduced with the p53^R175H^ virus, while AU565 cells were transduced with HLA-A*02:01 virus. Following a 48-hour incubation at 37°C, transduced cells were selected in the medium supplemented with 1 μg/mL puromycin (ST551-10mg, Beyotime, Shanghai, China) for 5 days. GFP-positive populations were then isolated by fluorescence-activated cell sorting (FACS) (Aria II, BD Biosciences) to establish stable cell lines designated as Saos-2-p53^R175H^ and AU565-HLA-A*02:01 (AU565-HLA). Additionally, the packaged virus with empty lentivectors were used to construct vector control (VC) cell lines, namely Saos-2-VC and AU565-VC. The expression of exogenous genes in these cell lines was confirmed through RT-qPCR analysis and Sanger sequencing.

**3.13 Evaluation of scDb-dependent cytotoxicity elicited by NK92mi-CD16a using confocal fluorescent imaging**

For confocal fluorescent imaging, target tumor cell lines (KMS26, Saos-2-VC, Saos-2-p53^R175H^, AU565-VC, AU565-HLA, KLE and KLE^tp53KO^) were collected and adjusted to a concentration of 1×10^6^ cells/mL. Then, 100 μL of the target cell suspension was seeded into 48-well plates containing fresh culture medium and incubated overnight at 37°C in a 5% CO_2_ incubator. Subsequently, effector NK92mi-CD16a (158V) cells were added to the wells at an effector-to-target (E:T) ratio of 10:1 in the presence of C33×CD16 scDb (5×10^-8^ M). The co-cultures were further incubated for 24 hours under the same conditions. The adherent cells were washed three times with 200 μL PBS then subjected to fluorescent staining using Cytotoxicity Assay Kit (C2015L, Beyotime, Shanghai, China). which employs Calcein-AM/PI staining to distinguish live cells from dead cells. Confocal fluorescent imaging was conducted to count dead cells (PI positive) and live cells (Calcein-AM positive) using ImageJ, which then was calculated the dead cell proportion.

**3.14 Fluorescence labeling of antibodies**

The purified anti-p53^R175H^/HLA-A*02:01 C33×CD16 scDb and the anti-HLA-A2 antibody (BB7.2) (343302, BioLegend, CA, USA) were labelled with Cy5 using sulfo-Cy5 (HY-D0821, MedChemExpress, NJ, USA). Briefly, sulfo-Cy5 was dissolved in dimethylsulfoxide (DMSO) at a concentration of 10 mg/mL to prepare the stock solution. The antibodies were concentrated to 5 mg/mL using an ultrafiltration unit (FUF053-25pcs, Beyotime, Shanghai, China) and then gently mixed with sulfo-Cy5 solution at a molar ratio of 10:1 (sulfo-Cy5 to antibody). Subsequently, the mixture was diluted with 1 M NaHCO_3_ to achieve a final antibody concentration of 2 mg/mL. The reaction tube was protected from light and incubated at room temperature with gentle shaking for 60 minutes. To enhance labeling efficiency, the tube was inverted every 10 minutes for 3 times. Meanwhile, Sephadex^®^ G-25 column was prepared according to the manufacturer’s instructions (G2580, Merck, NJ, USA). The equilibrated column was loaded with the reaction mixture and briefly spun for 10 seconds. The purified Cy5-labeled antibodies were collected in the outer tube and applied in subsequent experiments.

**3.15 Assessment of the potential for cytokine release syndrome (CRS)**

The PBMCs (from healthy donor) were adjusted to 2×10^6^ cells/mL. Then 100 μL of PBMCs were added to 96-well plates containing 100 μL culture medium in the presence of 10 μg/mL Fab control (Fab of C33-Ab without Fc fragment), 10 μg/mL C33×CD16, 0.5 μg/mL lipopolysaccharide (LPS) (L8880, Solarbio, Beijing, China), 1 μg/mL TGN1412 (HY-P9975, MedChenExpress, Shanghai, China). After 24 hours incubation, the PBMCs were centrifuged at 300 g for 5 minutes. The culture supernatants were collected and quantified the cytokine secretion level using ELISA.

**3.16 Animals**

All animal experiments were performed in accordance with the institutional guidelines for the care and use of laboratory animals. All mice were under constant surveillance by professional technicians throughout the course of treatments and experiments, and an external veterinary expert dedicated to animal welfare. All mice were housed in a specific-pathogen-free (SPF) environment with a regulated 12-hour light-dark cycle, maintained at a temperature range of 18-23°C and a humidity level of 40-60%. The mice had unrestricted access to a standard diet and water. Mice were euthanized upon reaching the experimental endpoint. All treatments were conducted after anesthetizing the mice with isoflurane.

**3.17 The evaluation of *in vivo* safety of C33×CD16 scDb**

Ten female MHC-humanized mice (NCG-HLA-A2.1) obtained from GemPharmatech (Nanjing, China), aged six to eight weeks, were randomly assigned to three groups and administered PBS (100 μL/dose), hFc-C33 (6 mg/kg/dose, 40 nmol/kg/dose), or C33×CD16 scDb (2 mg/kg/dose, 40 nmol/kg/dose) *via* intraperitoneal (IP) injection every 3 days from day 6 to assess long-term toxicity. Body weight was recorded from day 0. One dose of NK92mi-CD16a (1×10^7^ cells) was injected on day 0, 30 respectively. At the end of the experiment, mice were euthanized and tissue samples of five organs (heart, liver, spleen, lungs, and kidneys) were extracted. Histological analysis was performed on five organs from the euthanized mice using hematoxylin and eosin (H&E) staining to assess tissue morphology and overall organ health.
